# How to Conduct Usability Testing of Medical Education Simulations: Methods Tutorial Using a Telemedicine Simulation for Maternal Care

**DOI:** 10.2196/102697

**Published:** 2026-09-09

**Authors:** Blake J Lesselroth, Alexander Douglas, Helen Monkman, Romaric Marcilly, Karalane Bellavia, Mataeo Anderson, Jacob Van Buren, Charles Parsons, Sonakshi Mohanty, Juell Homco, Morgan Richards, Karen P Gold

**Affiliations:** 1 Department of Medical Informatics School of Community Medicine University of Oklahoma - Tulsa Tulsa, OK United States; 2 School of Health Information Science University of Victoria Victoria, BC Canada; 3 Department of Internal Medicine Oklahoma State University Tulsa, OK United States; 4 Univ. Lille, CHU Lille, ULR 2694 - METRICS: Évaluation des technologies de santé et des pratiques médicales, F-59000 Lille France; 5 Inserm, CIC-IT 1403, F-59000 Lille France; 6 University of Oklahoma Polytechnic Institute University of Oklahoma Tulsa, OK United States; 7 Department of Obstetrics and Gynaecology School of Community Medicine University of Oklahoma - Tulsa Tulsa, OK United States; 8 School of Community Medicine University of Oklahoma - Tulsa Tulsa, OK United States; 9 Department of Computer Science and Design University of California San Diego San Diego, CA United States

**Keywords:** human factors engineering, maternal health services telehealth simulation, medical education, simulation testing, simulation training, telehealth competencies, telemedicine, usability testing, user-centered design

## Abstract

**Background:**

Clinical educators regularly use simulation-based education to help learners develop clinical reasoning, procedural skills, and communication strategies. However, differences between simulated and live clinical environments, such as missing case details or workflow interruptions, can reduce realism and divert learners’ attention from the intended learning goals. While published design guidelines recommend pilot testing simulations, they do not explain how to systematically identify usability problems. Human factors evaluation methods can address this gap by identifying issues before piloting and providing design insights.

**Objective:**

This tutorial describes our usability testing protocol for evaluating educational simulations before piloting with learners. Using a telemedicine maternal care simulation as an example, we show how mixed methods usability testing can identify technical, workflow, and assessment-related issues in simulation design.

**Methods:**

We recruited obstetricians and family practitioners to test our simulation. To measure multiple usability dimensions concurrently, we used several published instruments, including an Agile Task Analysis (ATA), the Single Ease Question (SEQ), an adapted System Usability Scale (SUS), the Satisfaction With Simulation Experience Scale (SSES), and the NASA (National Aeronautics and Space Administration) Task Load Index (NASA-TLX). We also developed a competency assessment rubric to measure telemedicine competencies. We recorded observations on the ATA while testers used a think-aloud protocol. We then debriefed testers and administered the posttest questionnaires. We summarized quantitative data using descriptive statistics and analyzed qualitative data using theory-based deductive coding with published usability heuristics.

**Results:**

Twelve testers identified 52 usability issues over 2 testing cycles; 15 issues required correction before piloting with learners. Revisions included changes to standardized patient scripts, synthetic patient data, and scoring rubric instructions. Testers assigned a median SEQ score of 6 or higher to 4 of the 6 workflow steps; most struggled with unfamiliar telemedicine tasks or completing a virtual physical examination. SUS scores ranged from 65 to 100. The median SUS score was 77.5, indicating above-average usability. The NASA-TLX subscale scores were highest for mental workload (median 60, IQR 56.3-63.8) compared with other subscales, such as physical or temporal workload (median 15.00, IQR 11.3-18.8 and median 20.00, IQR 11.3-43.8, respectively), suggesting the simulation imposed a high mental demand. The overall SSES score of 43.5 out of 50 suggested faculty testers thought the simulation offered valuable learning opportunities.

**Conclusions:**

Following usability testing, we successfully piloted the simulation with 23 obstetrics and family medicine residents without encountering any implementation issues. Incorporating usability evaluation methods throughout the simulation development life cycle helped identify and mitigate design problems that might otherwise limit simulation fidelity and instructional effectiveness. Our tutorial showcases a modular mixed methods approach that can be adapted to study a range of simulation types and research questions related to workflow, technology, and learning objectives.

## Introduction

### Background

Health professional programs frequently use simulation-based training as an instructional strategy to introduce new concepts, enrich learning, and provide trainees with feedback [1,2]. Educational simulations are faculty-designed instructional methods that provide learners with realistic, immersive clinical situations to develop decision-making skills [1]. The simulation may include human actors representing specific roles (eg, standardized patients [SPs] and health care professionals) or technologies that mimic people and the environment (eg, augmented reality [AR], virtual reality [VR], AI, and manikins). Simulations are an important bridge between the classroom and the bedside, providing a safe yet realistic environment for trainees to learn material, test strategies, and develop reasoning skills before real-world practice [3]. Educators typically design simulations around scenarios with a clinical challenge and a normative workflow. The scenarios, however, should be flexible and responsive, allowing learners to explore, adapt, and improvise [2,4]. It is also critical that simulations include context-sensitive feedback or guided reflection informed by educational theory and learning objectives [2].

To meet instructional objectives, experts and professional societies have published best-practice recommendations for simulation development [2,5-7]. These recommendations are largely conceptual and lack the granularity needed to guide design, testing, and implementation. Conceptual approaches to design alone do not ensure simulation quality [8,9]. It can be challenging to preserve realism and consistency given the many variables affecting case fidelity and the achievement of learning objectives [4]. Simulationists must ensure the accuracy of the scenario, environment, and digital or practical assets, such as medical equipment and synthetic patient data (ie, ecological validity) [9]. Educators must evaluate learners in ways that align with learning objectives (ie, content validity) [4,10]. Finally, learners need personalized feedback addressing the ability under assessment (ie, construct validity) [2,10]. Optimizing validity often requires educators to limit the number and types of discrepancies between the simulation and real-world clinical practice [11,12].

Discrepancies between the simulation and the real world, including design omissions (eg, incomplete scenarios, data, or objectives) or equipment issues, can limit the simulation’s usability. These problems can consume cognitive resources that learners would otherwise apply to pedagogical tasks [11]. It therefore behooves developers to rigorously test the simulation from each person’s perspective (eg, the learner, the instructor, and SPs) and correct usability flaws before piloting with intended learners [4]. We authored this tutorial to share our methods and demystify the testing process.

### Problem Statement

Simulation development guidelines recommend rehearsing cases with representative learners and collecting feedback from content experts, colleagues, technical teams, and participants [2,4,8,9]. Yet simulation guidelines rarely include methods for testing design features or collecting performance data before piloting [2,4,8,13]. Recently, human factors researchers have published reports trying to address this methodological gap by applying usability measurement techniques [8,14,15]. The ISO defines usability as the “extent to which a system, product or service can be used by specified users to achieve specified goals with effectiveness, efficiency, and satisfaction in a specified context of use” [16]. Utility is what a system allows users to do, whereas usability is how easily and efficiently users can access and operate these functionalities. Together, these qualities contribute to the overall user experience (ie, “users’ perceptions and responses resulting from the use and/or anticipated use of a system, product or service”).

Usability testing is an evaluation method in which users interact with a product under realistic conditions to determine how effectively, efficiently, and satisfactorily a set of tasks can be completed [17]. Da Silva and Dubrowski [8], for example, used mixed methods to improve 2 simulations over 5 iterations, demonstrating how usability testing findings can inform revisions. Still, their approach required large numbers of learners and relied, in part, on learner self-reports—an often unreliable proxy for educational effectiveness [18]. We outline here a pragmatic strategy and toolkit to address these limitations.

Our team at the University of Oklahoma designed a series of simulations to teach telemedicine competencies to residents providing online maternal health care. We adapted published usability methods to create a simulation evaluation protocol. Our protocol had 2 aims: to identify technical and content issues requiring immediate correction and to gauge the completeness and difficulty of the clinical tasks. We focused on the following three usability issue types: (1) technology failures that could disrupt workflow; (2) missing case details, patient-script language, or synthetic clinical data crucial for medical reasoning; and (3) faculty scoring guide instructions that might be ambiguous or difficult to apply.

### Tutorial Aims and Goals

Our high-level aim with this tutorial is to provide medical educators, human factors experts, and simulationists with tools and instructions for usability testing educational simulations. Our learning objectives are to (1) explain how we adapted usability methods for this context, (2) share our data collection instruments with readers to facilitate replication (Multimedia Appendices 1-8), and (3) demonstrate our methods with a use case to illustrate how we applied our results (Textbox 1).

**Tutorial learning objectives.**

**After reading this tutorial, readers should be able to do the following:**

- Describe how to apply usability testing frameworks when designing instructional medical simulations.
- Adapt published usability testing methods and instruments to test educational simulations.
- Explain how to interpret usability findings to improve simulation consistency and teaching effectiveness.

While the objectives for usability testing are straightforward, we recommend articulating a priori research questions to guide protocol design and measure selection. Readers replicating our methods will likely formulate research questions relevant to their simulation. For our study, we had the following 3 questions:

- What failure modes interfere with the function of the simulation?
- Can faculty evaluators use the scoring guide to measure learner performance?
- Is the simulation difficulty appropriate for the intended learners?

## Methods

### Theoretical Framework: Usability and User Experience

Usability and user experience are not intrinsic properties of a product or a simulation; instead, they are emergent properties of the work system, arising from the interaction of multiple elements: technology, learners, instructors, tasks, environment, and organizational context [12,19]. This holistic perspective emphasizes that evaluating usability requires examining the entire work system, not the interface or equipment in isolation. Consequently, usability evaluation of simulations should aim to replicate realistic conditions regarding participants, task complexity, environmental constraints, and instructor support. Regarding educational simulations, this means evaluating not only the tools and technology but also faculty roles, faculty-learner interactions, and scenario details [20].

It is important to clarify how simulation usability can influence learners’ performance. Human-computer interaction shows that poor user experience increases the likelihood of errors [21-23]. Usability problems add cognitive load, forcing users to dedicate attention, working memory, and problem-solving capacity to compensate for suboptimal design. As a result, learners confronted with unintentional simulation design problems use cognitive resources to manage these problems rather than engaging with the learning objectives. Usability issues can reduce participant engagement, hinder learning, and diminish the perceived credibility of the program [12]. Hence, while simulation guidelines recommend piloting with learners to uncover problems, we believe usability issues should be addressed before piloting or implementation [2,6].

We needed to solve several unique problems in educational simulations that are atypical for usability testing of medical devices or software. First, we needed to calibrate scenario difficulty to participants’ experience level (eg, residents) and expertise (eg, obstetrics and gynecology [OB/GYN], family medicine). Second, faculty observers needed to evaluate learners for specific telehealth competencies to identify skill gaps. Third, we needed to include built-in challenges for the learner. Thus, we needed faculty participants to serve as observers to provide feedback on the scoring guide while gathering tester feedback on the simulation.

**Figure 1:**
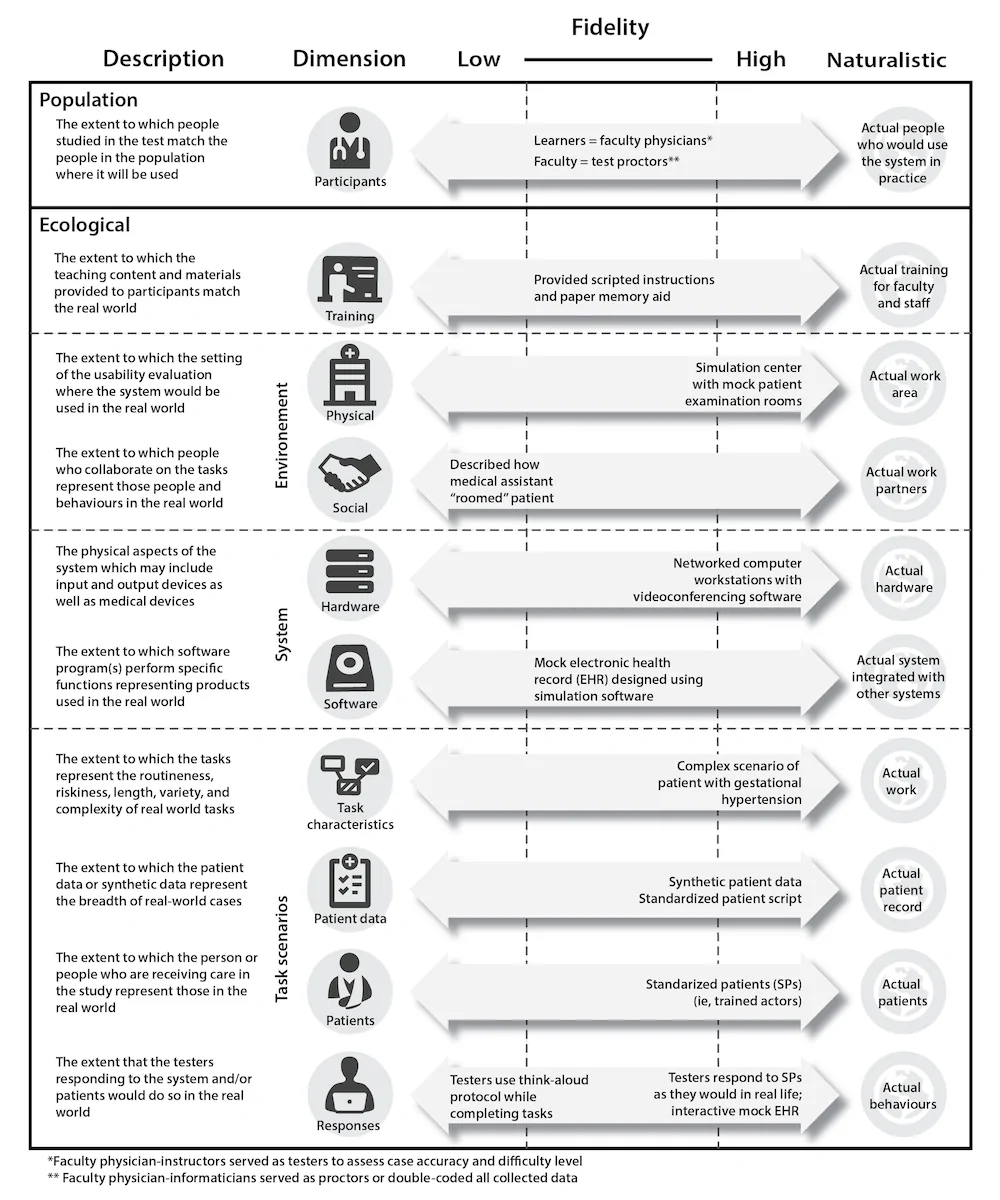
External validity framework for the application of usability testing in health. We adapted the figure to illustrate fidelity levels on a continuum for each dimension of our educational simulation.

The theoretical framework informing our current approach is based on a model of external validity we created for usability evaluations of health information technology (Figure 1) [20]. External validity comprises (1) population validity (ie, the extent to which the sample represents the population) and (2) ecological validity (ie, the extent to which the setting matches the real world). While both types of validity are important, the fidelity level for each dimension depends on the evaluation objectives [12].

When selecting the degree of ecological validity, we needed to consider learner attributes, presimulation didactics, the clinical environment (ie, physical and social), the technology (ie, hardware and software), and the scenario (ie, task characteristics, patient data, patients, and responses). Figure 1 [20] shows how we mapped model constructs to our use case. Poor usability arising from interactions between dimensions could manifest as unclear instructions, confusing interface layouts, incomplete patient scripts, mismatched equipment, or poorly structured task sequences. We chose to test our simulation with faculty rather than with learners. This was an intentional choice to assess case accuracy and to determine whether faculty believed the tasks were appropriately calibrated for resident learners.

### Use Case for Testing: A Telehealth Simulation for Residents Providing Maternal Care

During the COVID-19 pandemic, organizations embraced virtual care to enact social distancing precautions without limiting access [24-29]. This expansion highlighted educational gaps; practitioners were uncomfortable providing online care, and few training programs offered telemedicine instruction [30-35]. In response to this emerging need, the Association of American Medical Colleges (AAMC) published a white paper outlining a framework for telemedicine training in the United States [36]. The AAMC identified 20 competencies across six domains: (1) patient safety, (2) access and equity, (3) communication, (4) data collection, (5) technology, and (6) ethical and legal requirements. Since publication, medical schools and residencies have endeavored to teach this material using readings, didactics, and simulations [35,37-39].

At the University of Oklahoma School of Community Medicine (OUSCM), the Department of Medical Informatics partnered with the Department of Obstetrics and Gynecology to develop a telemedicine training program for maternal care [40]. Designed for OB/GYN and family and community medicine (FCM) residents, our simulations combined telemedicine instruction with vignettes managing maternal care conditions [41-43]. By training future physicians to provide online care to expectant mothers, we hoped to close a supply-demand mismatch contributing to local maternal care “deserts” [40,44-49].

Our educational program created the need—and the opportunity—to conduct usability tests of simulations before piloting with residents. The technology-forward nature of telemedicine inspired our team to adapt usability methods typically applied to health information technology. Although we have published preliminary reports describing individual tools, this is the first tutorial comprehensively describing our usability testing methods, findings, and downstream piloting experiences [41-43]. For illustrative purposes, we demonstrate our methods using a simulation of a fictional patient presenting for a telemedicine encounter to manage gestational hypertension.

### Study Setting and Research Team

Our research team conducted all testing at our university’s simulation center in Tulsa, Oklahoma, United States. The team included 2 obstetricians (MR, KB) to provide subject-matter expertise, 4 informaticians (BJL, HM, RM, and JH) and 2 data analysts to design the test protocol (MA and SM), a proctor to supervise each test and code the data (MA or CP), and at least 1 additional observer (typically another member of the research team) to double-code observations. The proctor and observers played the role of faculty during simulation testing. The team also included an SP and a simulationist (KB). The simulationist is an expert in simulation design, SP education, and script writing. The simulation center was equipped with mock clinic rooms, remote monitoring stations, simulation software, and computers with videoconferencing software. Because this was a telemedicine simulation, our SPs connected to the simulation center remotely via videoconference.

To recreate our described approach, we recommend the following resources: a dedicated space with internet access, a trained team member to serve as the SP, videoconferencing software with recording capabilities, and a proctor for the session. We also recommend recruiting team members to observe and double-code findings. We found it helpful to have trained faculty on-site to debrief testers. While the examination rooms in our center increased the ecological validity of our testing, they were not required.

### Data Collection Instruments

#### Overview

In this section, we describe each data collection instrument and our rationale for its inclusion (Table 1). Multimedia Appendices 1-8 provide instrument examples; Multimedia Appendices 9 and 10 are examples tools used for our specific simulation. We sought validated questionnaires to gather cross-sectional data on multiple usability dimensions. In our case, a “minimally viable product” for an educational simulation needed to satisfy three requirements: (1) the learner must be able to complete the scenario without interruption, (2) the learner must be able to review synthetic patient data needed for a clinical assessment, and (3) the faculty must be able to calculate competency scores for each learner.

**Table 1:** List of testing instruments used in our usability assessment protocol. We adapted usability dimensions and definitions from the ISO [16], Quesenbery [50], and Davis [51]. All instruments are available in Multimedia Appendices 1-9.

Instrument	Data collected	Measure type	Usability dimension	Usability dimension definition
Demographic screener (Multimedia Appendix 1)	Tester demographics, technology literacy, and attitudes and perceptions	Qualitative, binary (yes/no), and ordinal (Likert-type)	N/A^a^	N/A
ATA^b^ (Multimedia Appendix 2)	Simulation step completion rates	Ordinal (trichotomous)	Effective (learner)	How completely and accurately learners can complete a task to reach their goals [50]
ATA (Multimedia Appendices 2, 7, and 8)	Usability issues	Qualitative	Error tolerance	How well a design prevents errors or helps users recover from mistakes [50]
SEQ^c^ (Multimedia Appendix 3)	Task difficulty	Ordinal (Likert-type)	Easy to learn	The degree to which a person believes using a system would be free of effort [50]
Telemedicine competency rubric (Multimedia Appendix 9)	Clinical task completion rates, and competency scores	Task count and competency score	Effective (faculty)	How completely and accurately faculty could score the telemedicine rubric
Adapted SUS^d^ (Multimedia Appendix 4)	User perceptions	Ordinal (Likert-type)	Ease of use	How intuitive the product is, ensuring learners can understand expectations with little to no additional instruction [51,52]
SSES^e^ (Multimedia Appendix 4)	Learner perceptions	Ordinal (Likert-type)	Satisfaction	How relevant, valuable, and supportive the simulation was for acquiring skills and knowledge [53]
NASA^-^TLX^f^ (Multimedia Appendix 5)	Cognitive load	Ordinal	Efficiency	The resources expended, including time, cognitive load, and effort in relation to the accuracy and completeness of goals [54]
Debrief interview guide (Multimedia Appendix 6)	Usability issues	Qualitative	Satisfaction	How pleasant, satisfying, and aesthetically pleasing the product is [16]
Debrief interview guide (Multimedia Appendix 6)	Usability issues	Qualitative	Fidelity	How completely and accurately the tasks align with real-world scenarios [11,55]

^a^N/A: not applicable.

^b^ATA: Agile Task Analysis.

^c^SEQ: Single Ease Question.

^d^SUS: System Usability Scale.

^e^SSES: Satisfaction With Simulation Experience Scale.

^f^NASA-TLX: NASA (National Aeronautics and Space Administration) Task Load Index.

#### Demographics Questionnaire

When considering population validity, it is helpful to create a user persona and recruit testers matching the persona’s characteristics. Learners are the target users for educational simulations and can provide useful feedback. While it may seem counterintuitive, it has been our experience that faculty can also provide useful feedback. They have the domain expertise to critique simulation fidelity, task complexity, the need for additional scaffolding, and alignment with learning objectives [4]. Hence, we recommend recruiting faculty testers first, followed by learners as time and resources permit. For all recruited participants, we gathered descriptive demographic data to share with stakeholders (eg, educational leadership, executive sponsors, collaborative partners, funders) when presenting findings. We therefore used a demographics questionnaire with 3 sections: (1) tester demographics, (2) a technology literacy assessment, and (3) knowledge and attitude items about telemedicine (Multimedia Appendix 1). We confirmed that all testers provided maternal medical care. We asked whether they regularly used a computer, mobile technology, or videoconferencing software. Finally, we included a short questionnaire covering prior experiences with telemedicine. For the latter section, we based the content domains on research exploring the intersection between clinician attributes and successful telemedicine encounters [56].

#### Agile Task Analysis

Agile Task Analysis (ATA) is a hybrid method that combines hierarchical task analysis (HTA) with qualitative observation during simulation testing (Multimedia Appendix 2) [57]. In a standard HTA, the researcher breaks complex activities into tasks and goals to understand how work is performed and what tools and information are required for each task [58]. Observations may include task lists, narrative details, workflows, and examples of tools, paper forms, or technology [59]. Borrowing from HTA, the ATA uses this framework to organize scenarios for simulation testing [57]. It also includes a data collection form that follows the scenario's normative workflow, allowing observers to record observations concurrently. Data can be quantitative or qualitative, including task completion rates, observed errors, usability issues, or user observations, such as quotes or design recommendations.

**Figure 2:**
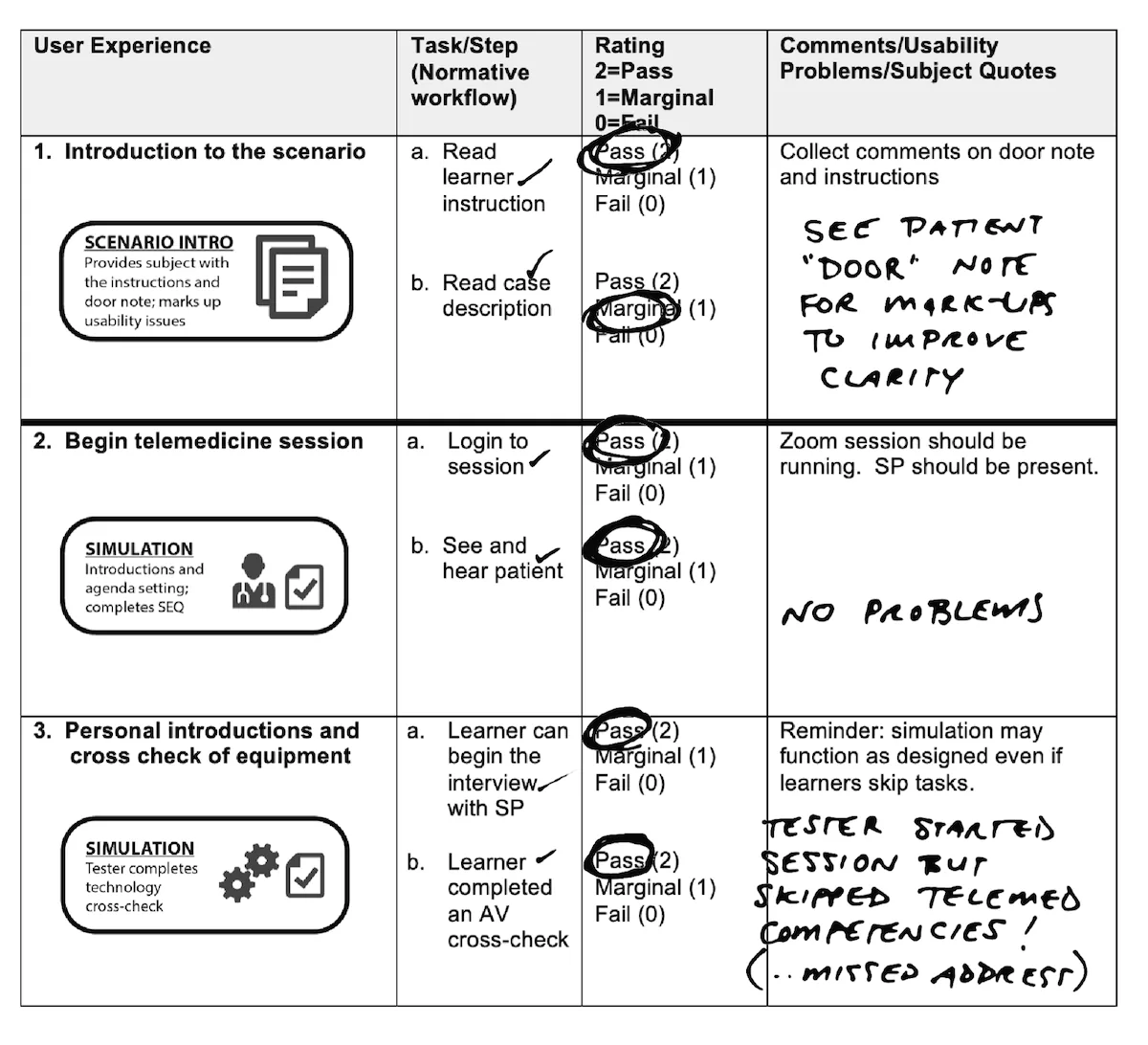
Excerpt from the Agile Task Analysis (ATA) form we used to evaluate our telehealth maternal care education simulation.

Researchers can modify the ATA based on the type of product or research questions. For example, the ATA can include screenshots of technology interfaces, informational handouts, or blueprints of the physical environment [57]. For this study, we listed six concept-level steps in our telemedicine workflow: (1) read learner instructions, (2) start a telemedicine session, (3) complete standard telemedicine tasks, (4) collect a patient history, (5) review examination and objective data, and (6) develop an assessment and plan (Figure 2 [57]). For each step, the research team tracked simulation performance using an ordinal trichotomous scale (ie, pass, marginal, and fail). We coded a step as “pass” if the tester completed it without assistance, “marginal” if the tester required proctor assistance, or “fail” if the tester encountered a critical fault in the simulation and needed to bypass the step entirely.

#### Telemedicine Competency Rubric

Educational simulations are often designed to challenge learners or to identify knowledge or skill gaps. A simulation step can work as designed even if the tester neglects a task or performs it incorrectly. We did not expect testers to complete every task. To differentiate simulation steps from telemedicine tasks, we included a telemedicine competency rubric (ie, faculty scoring guide) in the ATA to record testers’ performance on telemedicine tasks during the simulation (Multimedia Appendix 2) [41].

Our telemedicine competency rubric was based on the AAMC white paper and operationalized measurement for 8 of the 20 listed telehealth competencies [36]. We selected 8 competencies for our simulation: 2 focusing on patient safety, 2 on communication skills, 1 on data collection, 2 on telehealth technologies, and 1 on ethical requirements. The AAMC white paper includes definitions for each competency but not context-specific details for clinical care or explicit measurement strategies. We were forced to create a novel rubric that organized each competency into short sets of related, contextually embedded, and directly observable tasks (Multimedia Appendices 2 and 9) [60]. We summed correctly performed tasks to calculate composite competency scores. We operationalized competency into 3 levels: entrustable, approaching entrustment, and not entrustable [60,61]. A learner was “entrustable” if faculty believed the learner could be entrusted to complete all activities within a content domain without requiring direct supervision. Faculty marked a learner as “approaching entrustment” if they believed the learner needed some supervision to complete related clinical tasks. The tester needed to complete at least half of the tasks within a domain to be approaching entrustment. A learner was “not entrustable” if faculty believed the learner must be directly supervised throughout the activity. The proctor marked a tester “not entrustable” if the tester completed fewer than 2 tasks within a telemedicine domain. As a first step toward establishing interrater reliability, we required multiple research team members to score each tester independently during data collection and then resolved discrepancies through discussion and consensus (details are provided in the Data Recording and Adjudication section).

#### Single Ease Question

The Single Ease Question (SEQ) is a 7-point Likert-type question administered immediately after a task, asking the tester to rate the task’s difficulty [23,62]. Tasks that score higher than 6 are considered “very easy” [63]. The SEQ offers insights that posttest questionnaires do not by dissociating individual steps from the overall user experience [64,65]. We included 1 SEQ after each of the 6 workflow tasks and 1 at the conclusion of the simulation (Multimedia Appendix 3).

#### Adapted System Usability Scale

The System Usability Scale (SUS) is a 10-item, 5-point Likert-type scale assessing users’ self-reported ease of use with a product [23,52,65,66]. Researchers convert respondents’ answers to a 100-point scale. Based on the distribution of scores across industries, an average score is 68 [52]. We adapted the SUS language to evaluate testers’ perceptions of a simulation rather than a technology (Multimedia Appendix 4). Prior studies of the SUS show that substituting the word “system” with a contextually appropriate product name or referent does not degrade the psychometric properties of the instrument [67].

#### Satisfaction With Simulation Experience Scale

The Satisfaction With Simulation Experience Scale (SSES) is an 18-item instrument used to evaluate learner satisfaction with educational simulations [53]. It measures learners’ engagement and perceptions of simulation effectiveness [53,68]. Although not a traditional usability measure, we used the SSES to capture faculty and resident perceptions of the simulation's educational value and treated it as a proxy for satisfaction. The SSES complements other usability measures (eg, SEQ, SUS, and think-aloud) but does not replace them. Items are grouped into 3 subscales: debriefing and reflection, clinical reasoning, and clinical learning. For this study, we adapted questions from the SSES clinical reasoning and clinical learning subscales, modifying the language for a telemedicine simulation (Multimedia Appendix 4).

#### NASA Task Load Index

To measure perceived simulation complexity, we administered the NASA (National Aeronautics and Space Administration) Task Load Index (NASA-TLX) after the simulation (Multimedia Appendix 5) [54]. The NASA-TLX was developed by NASA to measure perceived workload and cognitive overhead associated with a set of related tasks [69]. There are 6 subscales: mental demands, physical demands, temporal demands, perceived performance, effort, and frustration [54]. Each dimension is scored on a 100-point scale. Although there are no formal cut points, studies suggest scores ≤30 indicate a low workload, scores between 31 and 50 indicate a moderate workload, and scores >50 indicate a high workload [54,69-71]. The original NASA-TLX requires participants to assign a weight to each subscale. Researchers often omit the weighting step and use raw scores [69]. We used unweighted scores in our analysis.

#### Debrief Semistructured Interview

We used a semistructured debriefing guide to gather testers’ final reflections (Multimedia Appendix 6). We designed our guide based on the Rapid Usability Evaluation method by Russ et al [72] and *Rocket Surgery Made Easy* [73] by Steve Krug. In both cases, the authors recommend asking probing questions during the observation session immediately following tasks and limiting the debrief to 1 or 2 open-ended follow-up questions. The researcher can use these questions to clarify usability issues or invite the participant to suggest changes for improving the design. We used questions and probes to explore the simulation’s difficulty, the root causes of usability issues, and opportunities to improve the learning experience. We recorded responses in our usability issue log.

### Testing Protocol

#### Overview

**Figure 3:**
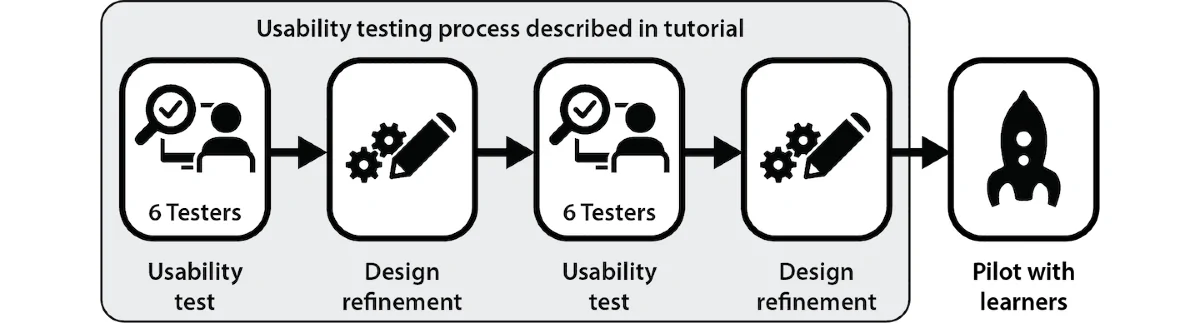
Testing and refinement strategy. We used new testers for each round. The research team, which included content experts, served as faculty observers.

This section describes our testing protocol (Figure 3). While our general approach can be adapted to a range of educational simulations, the specifics of each simulation will dictate the number of testing cycles. We organized testing into sprints, gathering feedback from 6 testers before iterating on the design. We conducted 2 rounds of simulation testing and revisions (ie, sprints) to refine the simulation. We used new testers for each testing cycle to avoid data contamination. We conducted 2 refinement sessions, once at the midpoint of testing and once before piloting with target learners. We avoided making refinements to the simulation between refinement sessions.

#### Recruitment

We used a purposive recruitment strategy, targeting faculty and residents to test the simulation and provide feedback on the scenario’s accuracy and fidelity. Faculty and residents were eligible for inclusion if they (1) held an appointment at our institution, (2) had completed or were participating in a residency in obstetrics or family medicine, and (3) maintained an active obstetrics practice. Residents in obstetrics and family medicine are required to see obstetrics patients in a continuity clinic during training; they practice under the license of their supervising attending. We contacted every eligible practitioner at our institution and recruited the first 6 volunteers for each testing round, aiming to balance the number of obstetricians and family practitioners.

#### Tester Orientation

**Figure 4:**
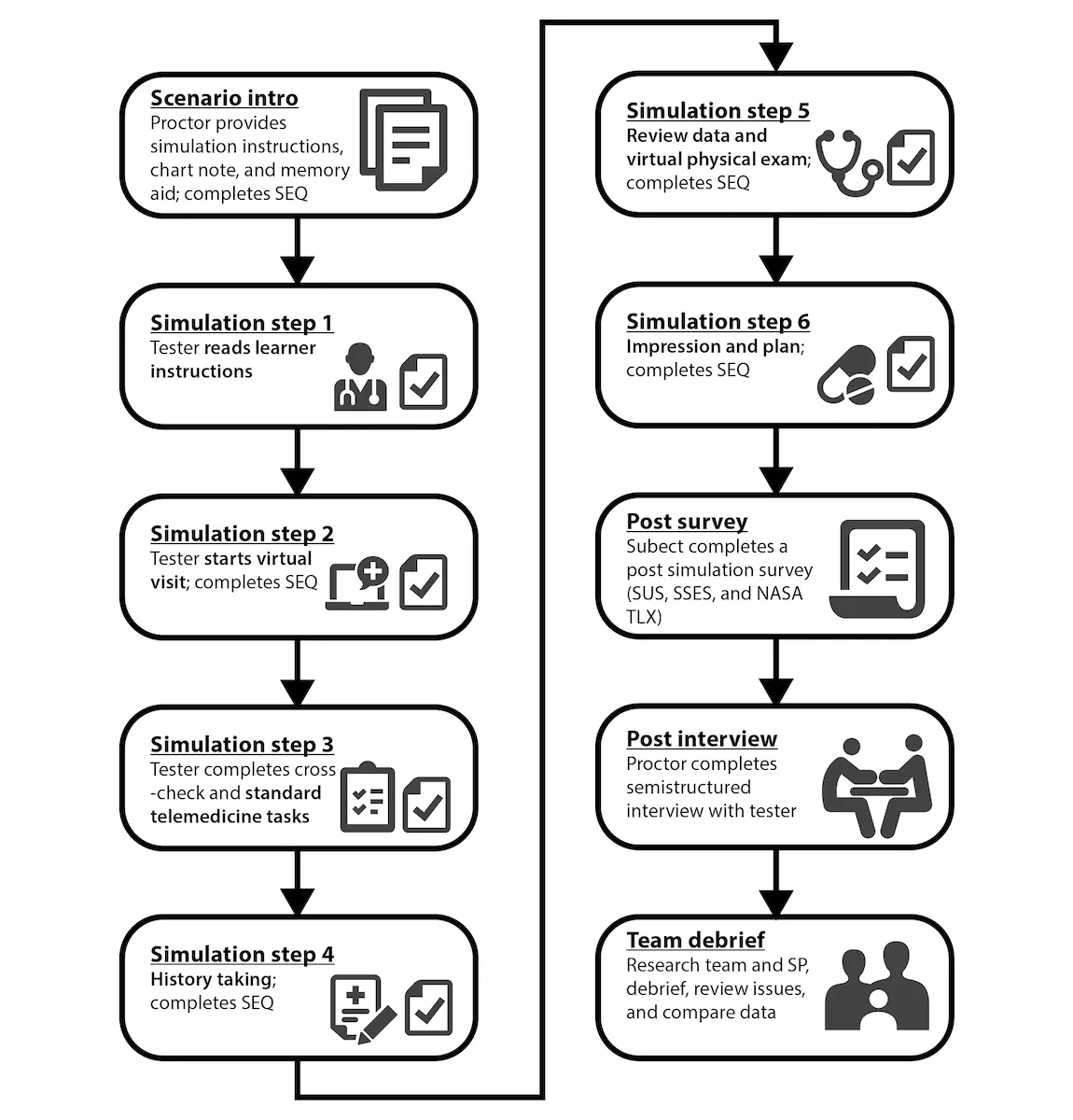
Schematic for the usability testing protocol. We conducted 2 rounds of formative usability testing before piloting with target learners. NASA-TLX: NASA Task Load Index; SEQ: Single Ease Question; SP: standardized patient; SSES: Satisfaction With Simulation Experience Scale; SUS: System Usability Scale.

**Figure 5:**
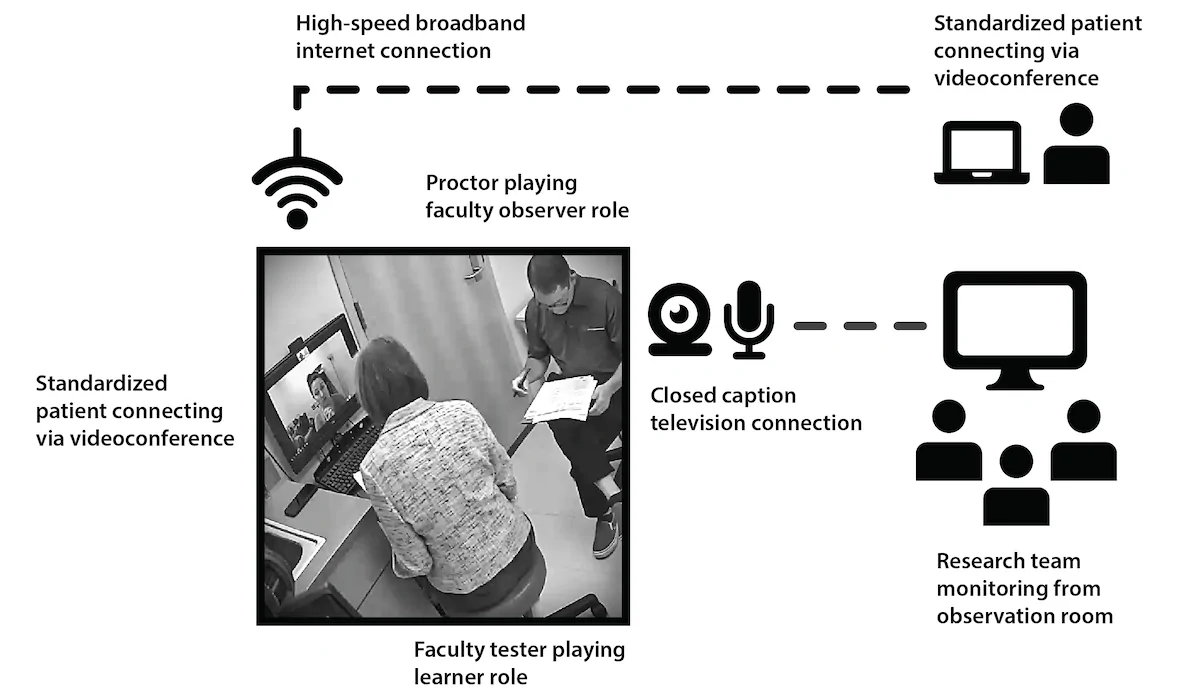
Setting and configuration for usability testing.

A member of the research team provided each tester with an overview of the study, including the curriculum's purpose and the high-level goals for the simulation (Figure 4). We secured verbal and written consent from each tester during the orientation to observe and record testing and tester insights. The demographic screening questionnaire included consent language and an explanation of how the data would be used. We entered data, excluding unique identifiers, into a spreadsheet for subsequent analysis. We did not include a telemedicine didactic before testing. We did not disclose scenario details, workflow expectations, or the competencies being evaluated. A proctor sat with the tester in a mock examination room with a computer workstation, microphones, and closed-circuit cameras (Figure 5). A proctor without clinical expertise can be advantageous as a neutral observer. Before starting the simulation, the proctor furnished the tester with the screening questionnaire. We adapted the Rapid Usability Evaluation method by Russ et al [72] and the methods outlined in *Rocket Surgery Made Easy* by Steve Krug [73]. The proctor read aloud a written script explaining the purpose of testing and the think-aloud technique (ie, testers verbalize their thoughts while interacting with the system and completing tasks) [74,75].

#### Simulation Testing

During the simulation, the tester used the computer workstation with videoconferencing software (Zoom; Zoom Communications) to complete a mock encounter with our SP [76]. We furnished the tester with a summary of the case: a pregnant woman living in a rural community and presenting at 20 weeks’ gestation for a routine follow-up appointment. The patient reported recording new high blood pressure values using a home digital blood pressure cuff.

In the first round, the proctor provided the tester with written case instructions, a mock clinical note with synthetic patient data, and a printed memory aid developed by the team to help with telemedicine tasks [77]. In the second round, rather than providing a mock clinical note, the tester reviewed synthetic patient data using a mock electronic health record (EHR) we designed with LearningSpace simulation software (Elevate Healthcare) [78]. We continued to give the tester written case instructions and our memory aid. The proctor did not provide technical instructions, additional medical information, or clinical feedback to the tester during the simulation. The other members of the research team watched the simulation on closed-circuit television and recorded observations simultaneously.

We expected the tester to establish rapport with the SP, troubleshoot technology problems, gather a history, review synthetic patient data, provide a clinical assessment, and communicate a treatment plan. The learner needed to gather enough clinical data to rule out preeclampsia, an obstetric emergency. During the simulation, the proctor recorded all observations on the ATA paper form. The proctor and observers recorded (1) whether each simulation step worked, (2) whether the tester had the opportunity to perform each competency-related task, (3) whether the tester performed the task successfully, and (4) whether the tester encountered any usability issues. Initially, the proctor asked the SEQ after each set of related tasks. However, we found this disrupted the simulation workflow and added cognitive overhead. Hence, the proctor asked all SEQs after the simulation.

#### Postsession Questionnaires and Semistructured Interviews

After the simulation, the proctor administered our postsession questionnaires (ie, SUS, SSES, and NASA-TLX) and conducted a semistructured interview (Multimedia Appendices 4-6). The interview provided an opportunity to explore areas of confusion, the root causes of usability issues, potential modifications to the simulation, and unmet educational needs.

#### Data Recording and Adjudication

For the rubric, the research team compared scores for each task and resolved discrepancies through discussion to reach consensus. When we identified vague or potentially confusing language, we revised the task description during the design and refinement phases. We did not calculate agreement statistics during testing. The Limitations section provides a more complete discussion of our rationale and next steps. We compiled these results into a “master” document. We then reviewed all qualitative data, including usability issues, observations, tester quotes, and interview responses. We summarized the qualitative findings in an issue log (Multimedia Appendices 7 and 8) for content analysis, coding, prioritization, solution ideation, and potential action.

### Simulation Piloting

After we completed all usability testing and simulation refinements, we piloted the entire workshop (ie, a telemedicine didactic session, telemedicine simulation with the competency rubric [Multimedia Appendix 9], and postsession questionnaire) with the residents. A detailed protocol for our workshop and simulation pilot falls outside the scope of this tutorial. We included a brief vignette of the pilot herewith (Textbox 2) to illustrate the downstream value of usability testing.

**Workshop and simulation vignette.**

A complete description of workshop piloting with residents is beyond the scope of this report. We intend to describe the implementation methods and residents’ scores in a companion manuscript. In this section, we include a short vignette of our piloting experience.

We conducted 2 separate pilots on different afternoons, 1 with 6 obstetrics and gynecology residents and 1 with 17 family and community medicine residents. All residents received a brief overview of the case, expectations for the simulated encounter, and a paper copy of the telemedicine memory aid. Residents used computer workstations in our testing center to interview a standardized patient and had online access to the mock electronic health record with synthetic patient data.

All participants completed the simulation without experiencing any unanticipated technical problems. Faculty were comfortable using the supplied evaluation rubric and had no difficulty providing individualized learner feedback after the simulation. Resident feedback on the simulation was generally favorable; most indicated the simulation provided a clear, safe, and constructive learning experience.

### Data Analysis

#### Quantitative and Statistical Analysis

We calculated descriptive statistics for quantitative data, including demographic characteristics, simulation step completion rates, competency scores, SEQ scores, SUS scores, SSES scores, and raw NASA-TLX scores. Given the small sample size and the nonparametric nature of the data, we calculated raw counts, medians, and IQRs. Given the formative nature of the analysis, we did not conduct an a priori power analysis or calculate comparative statistics.

#### Qualitative Analysis

While a formal qualitative analysis is not always critical for pragmatic usability testing, we found it helpful for clarifying the root causes of issues, brainstorming potential solutions, and prioritizing fixes. Using coding methods described by Saldana, we conducted a content analysis of qualitative data gathered during the simulation and tester debriefing interviews [79]. We used simultaneous coding to assign a categorical usability code, a valence (ie, “positive” or “negative”), and a priority for action. For categorical codes, we used a deductive, theory-based coding strategy based on usability heuristics published by Zhang et al [80] (Multimedia Appendix 7) [21,81]. At least 2 members of the research team independently coded each finding. We then compared codes for concordance and resolved discrepancies through discussion. We assigned a provisional code with emergent supplementation to any findings that could not be classified according to the usability heuristics published by Zhang et al [80]. All codes and findings were recorded in a usability issue log (structural codes are provided in Multimedia Appendix 7; a sample log is provided in Multimedia Appendix 8).

### Ethical Considerations

The testing described in this tutorial was reviewed by the University of Oklahoma Institutional Review Board and classified as a quality improvement initiative associated with normal educational activities. Nevertheless, we collected verbal and written consent from each tester to document the testing.

## Results

### Tester Demographics

We recruited 12 clinicians for usability testing (first round: 4 OB/GYNs and 2 FCMs; second round: 2 OB/GYNs and 4 FCMs). Table 2 provides demographic details.

**Table 2:** Tester demographic information and technology literacy.

Characteristic			Value		Mean (SD)		Median (IQR)
**Demographic**							
	Age (years), range	25-60		38.9 (12.1)		37.0 (28.5-45.0)	
	Work experience (years), range	1-30		8.8 (11.1)		3.5 (1.0-10.5)	
	Specialty, n	OB/GYN^a^=6; FCM^b^=6		—^c^		—	
**Technology literacy, n**							
	Use computer once a week	Yes=12; no=0		—		—	
	Use portable technology	Yes=12; no=0		—		—	
	Use videoconferencing	Yes=12; no=0		—		—	
**Attitudes and perceptions, n**							
	Previously observed a telehealth encounter	Yes=11; no=1		—		—	
	Previously provided telehealth care	Yes=10; no=2		—		—	
	Previously received telehealth care	Yes=5; no=7		—		—	
	Received telemedicine instruction	Yes=4; no=8		—		—	
	Good understanding telemedicine practice	—		3.9 (0.70)		4.0 (4.0-4.0)	
	Familiar with types of technology	—		3.8 (0.8)		4.0 (3.8-4.0)	
	Think telemedicine is a good option	—		3.7 (1.3)		4.0 (4.0-4.0)	
	Will likely use telemedicine in practice	—		3.9 (0.8)		4.0 (4.0-4.0)	

^a^OB/GYN: obstetrics and gynecology.

^b^FCM: family and community medicine.

^c^Not applicable.

### Overall Results

Table 3 summarizes all usability measures, with key findings highlighted. We include detailed results for each measure in the following sections. The ATA identified 4 problematic steps in round 1 that we resolved by round 2. Across both rounds, we identified and addressed 15 critical usability issues. The virtual physical examination received the lowest SEQ ratings. During round 2, testers reported a median SUS score of 77.5 (IQR 73.1-80.0) the SUS and a median SSES score of 42 (IQR 41.0-46.75) out of a possible 50. The median NASA-TLX mental demand score in round 2 was 60.00 (IQR 56.3-63.8); a score >50 indicates a high workload. Proctors could not score all testers with the rubric during the first round. After modifying the rubric language during the first refinement phase, proctors successfully scored every tester.

**Table 3:** Summary table of quantitative results across instruments.

Usability dimension, instrument, and measured item				Frequency, n/N (%)		Median (IQR)		Maximum possible value
**Effectiveness**								
	**Agile Task Analysis**							
		Total number steps completed successfully	165/180 (92)		—^a^		—	
		Total number steps partially completed	11/180 (6)		—		—	
		Total number of failed steps	4/180 (2)		—		—	
**Error tolerance**								
	**Agile Task Analysis**							
		Number of usability issues	52		—		—	
		Number of critical usability issues	15/52 (29)		—		—	
**Easy to learn**								
	**Single Ease Question**							
		Reviewing materials	—		6.00 (6.00-6.25)		7.00	
		Starting encounter	—		6.00 (4.75-7.00)		7.00	
		Standardized telemedicine tasks	—		5.50 (5.00-6.25)		7.00	
		History collection	—		6.50 (5.00-7.00)		7.00	
		Virtual physical examination	—		4.50 (2.75-6.00)		7.00	
		Diagnosis and plan	—		6.00 (5.00-6.50)		7.00	
		Overall encounter	—		5.00 (5.00-6.00)		7.00	
**Ease of use**								
	**Adapted System Usability Scale**							
		Total score round 1	—		73.80 (72.63-80.50)		100.00	
		Total score round 2	—		77.50 (73.13-80.00)		100.00	
		Total score overall	—		75.00 (71.88-81.25)		100.00	
**Satisfaction**								
	**Satisfaction With Simulation Experience Scale**							
		Total score round 1	—		44.50 (39.5-45.0)		50.00	
		Total score round 2	—		42.00 (41.00-46.75)		50.00	
		Total score overall	—		43.50 (40.5-45.25)		50.00	
**Workload**								
	**NASA^b^ Task Load Index**							
		Physical demand round 1	—		7.50 (5.00-17.50)		100.00	
		Temporal demand round 1	—		12.50 (6.25-22.50)		100.00	
		Frustration level round 1	—		22.50 (10.00-42.50)		100.00	
		Perceived performance round 1	—		32.50 (26.25-35.00)		100.00	
		Mental demand round 1	—		37.50 (21.25-57.50)		100.00	
		Effort (mental and physical) round 1	—		42.50 (27.50-57.50)		100.00	
		Physical demand round 2	—		15.00 (11.30-18.80)		100.00	
		Temporal demand round 2	—		20.00 (11.30-43.80)		100.00	
		Frustration level round 2	—		25.00 (10.00-43.80)		100.00	
		Perceived performance round 2	—		32.50 (21.30-43.80)		100.00	
		Mental demand round 2	—		60.00 (56.30-63.80)		100.00	
		Effort (mental and physical) round 2	—		55.00 (46.30-63.80)		100.00	
		Mental demand overall	—		57.50 (23.75-61,25)		100.00	
		Effort (mental and physical) overall	—		50.00 (32.50-60.00)		100.00	
**Effectiveness**								
	**Competency rubric^c^**							
		Incorporates telehealth	12/12 (100)		—		—	
		Attends to environment	11/12 (92)		—		—	
		Identifies and uses equipment	12/12 (100)		—		—	
		Develops rapport	12/12 (100)		—		—	
		Obtains and uses history	12/12 (100)		—		—	
		Troubleshoots technology	12/12 (100)		—		—	
		Prepares for escalating care	12/12 (100)		—		—	
		Adheres to professional requirements	10/12 (83)		—		—	

^a^Not applicable.

^b^NASA: National Aeronautics and Space Administration.

^c^Indicates the number of learners successfully assessed.

### ATA Results

#### Simulation Step Completion Counts

**Figure 6:**
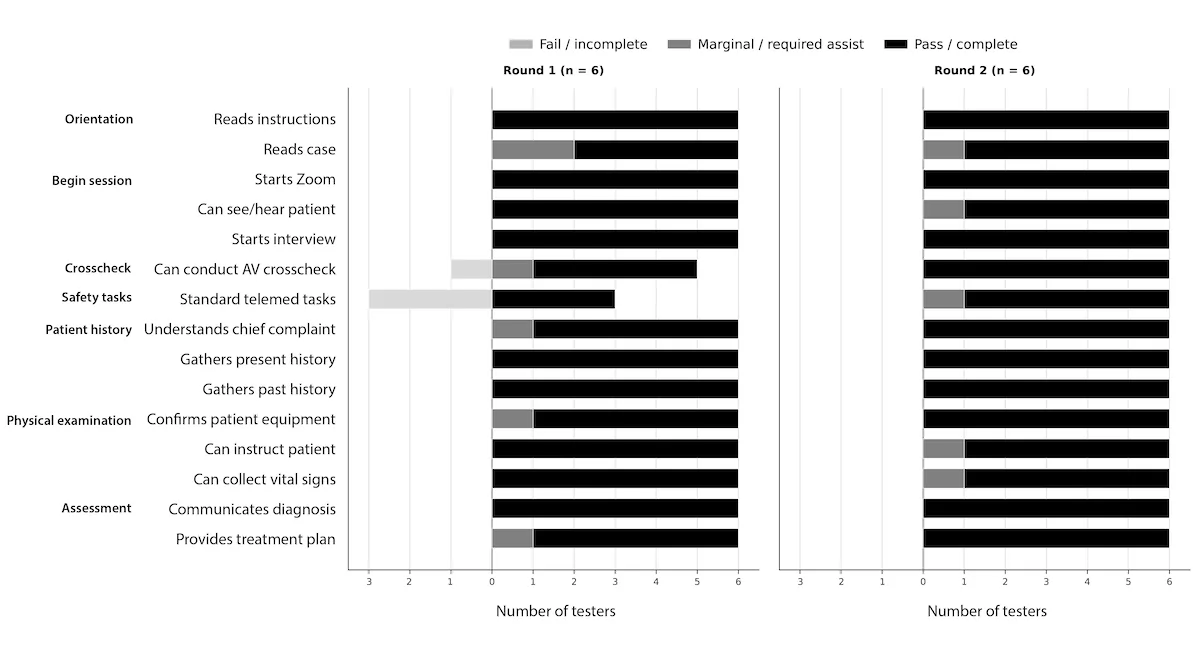
Simulation step completion counts. We show scores for each testing round, separated by one refinement session. AV: audiovisual.

Figure 6 shows simulation completion rates organized by workflow step. While most testers completed the steps without difficulty, not every tester completed every step. In the first round, testers skipped standard telemedicine patient safety tasks (eg, confirming the patient’s physical location). This issue improved during the second round after we included a mock EHR with synthetic data and visual cues (eg, patient address and phone number fields).

#### Usability Issues

We logged 52 usability findings across both testing rounds; 15 required corrections before piloting (Tables 3 and 4). We identified issues with the SP script, gaps in synthetic patient data, deviations in the simulation workflow, and problems with the competency rubric language. New issues emerged after the first refinement session due to updates to materials and assets (eg, the addition of a mock EHR).

**Table 4:** Excerpt from our usability issue log illustrating a sample of usability issues and remediation strategies organized according to the usability heuristics schema [80].

Test number	Finding	Code^a^	Valence	Priority	Fixed?	Intervention
7	The tester expected to see a flowsheet of blood pressure values in the mock electronic health record	Match	Negative	High	Yes	Added a flowsheet with additional blood pressure values to the LearningSpace software
8	Need to include photographs of physical findings, such as leg edema	Feedback	Negative	High	Yes	Gave the SP^b^ printed photographs of physical examination findings sourced from licensed stock photography

^a^Code: usability heuristic code.

^b^SP: standardized patient.

Usability issues tended to fall into the following four heuristic categories: (1) user feedback, (2) match between the system and the real world, (3) error prevention, and (4) system visibility. For feedback, testers asked to see the patient’s leg to check for edema. We provided SPs with photographs of ankle edema to show on camera. To align the system with the real world, testers requested a flowsheet of blood pressure values, which we added to our mock EHR. As an example of an error-prevention issue, faculty needed a clear deadline in the task definition to score learners scheduling follow-up laboratory tests. We added “within 1 week” to the rubric. As an example of a visibility issue, faculty could not tell when learners fabricated information to preserve the illusion of the simulation (eg, naming a fictional laboratory). We therefore added a question to the SP script that the learner would have to research (ie, the location of the laboratory nearest the patient’s home).

### SEQ Results

**Figure 7:**
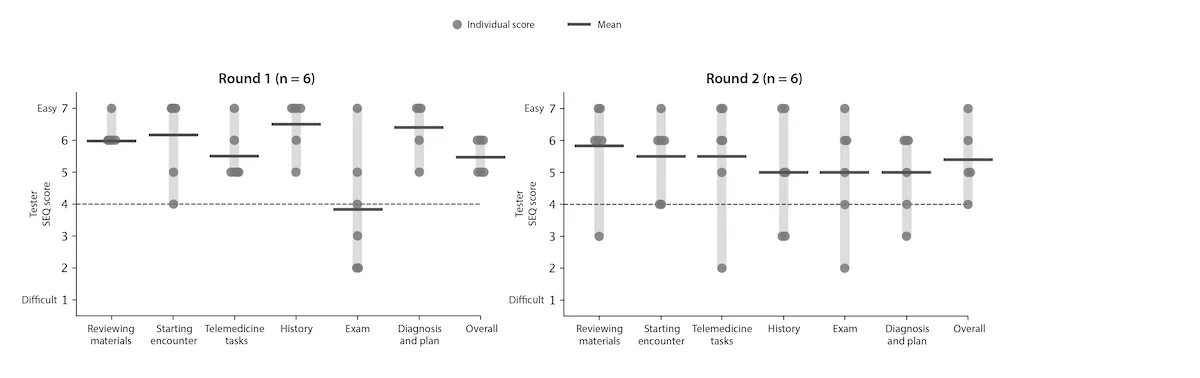
Single ease question results for both testing rounds. We show scores for each testing round, separated by one refinement session.

Most testers found the simulation instructions and steps easy to understand (Figure 7 [55]). Testers assigned a median SEQ score of 6 or greater to 4 of the 6 components. Testers said completing a virtual physical examination (median 4.50, IQR 2.75-6.00) was the most challenging step.

### SUS Results

**Figure 8:**
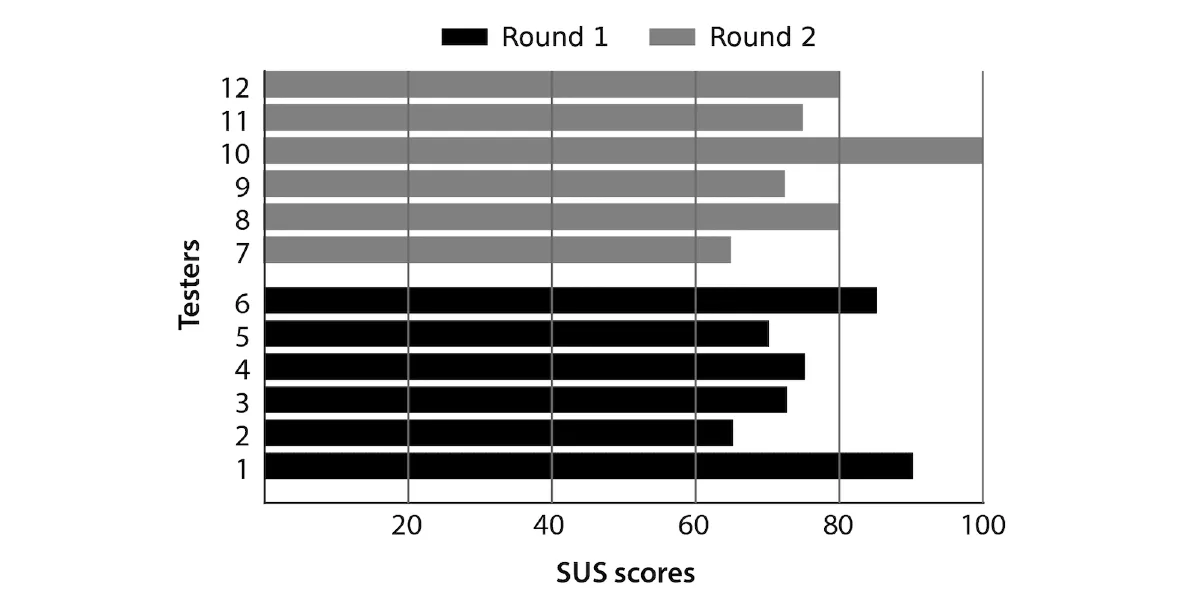
System Usability Scale (SUS) scores separated by testing rounds. We show scores for each testing round, separated by one refinement session.

Testers reported some challenges with ease of use and learnability (Figure 8 [52]). SUS scores ranged from 65 to 100. For round 1, respondents’ median score was 73.80 (IQR 70.63-82.50); for round 2, the median score was 77.50 (IQR 73.13-80.00). The inclusion of a mock EHR in round 2 may account for the increase in the median SUS score.

### SSES Results

**Figure 9:**
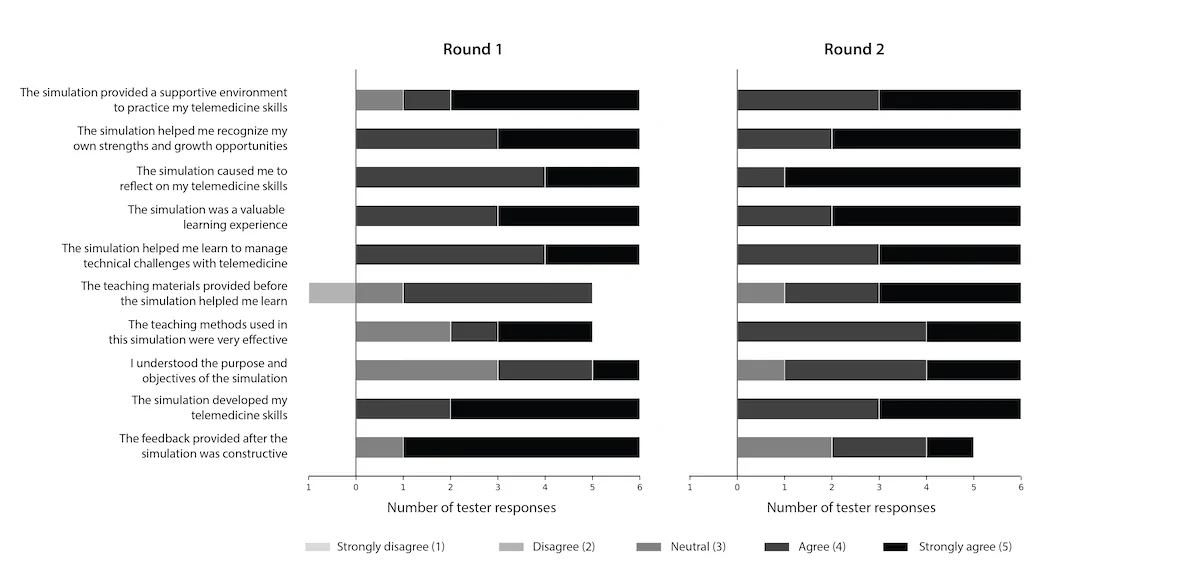
Satisfaction With Simulation Experience Scale scores. We show scores for each testing round, separated by one refinement session.

The SSES scores indicate that most testers considered the simulation a valuable learning experience (Figure 9 [53]). One tester disagreed with the statement that the simulation tested their clinical abilities.

### NASA-TLX Results

**Figure 10:**
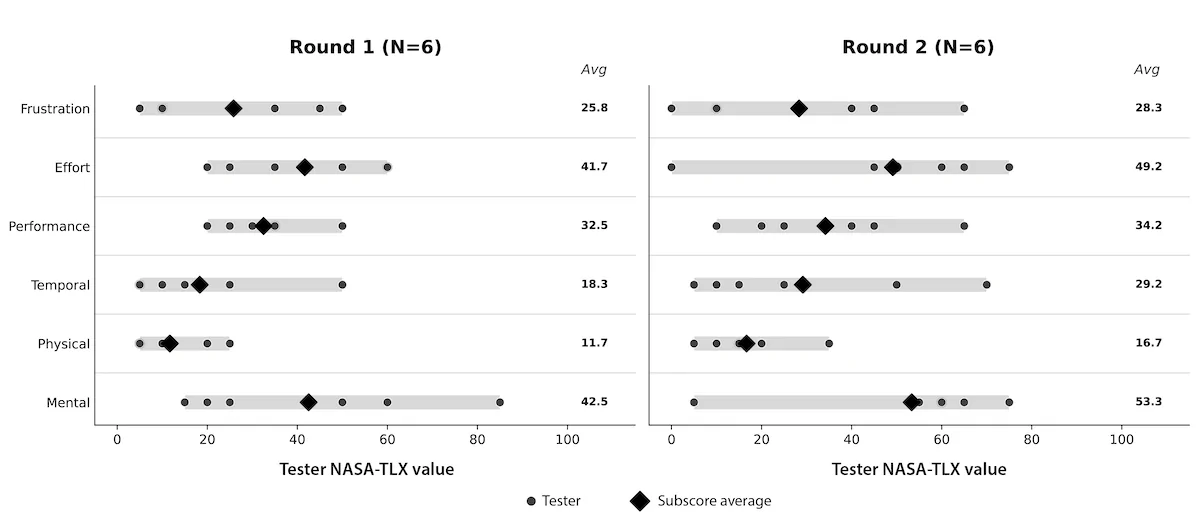
Unweighted NASA Task Load Index (NASA-TLX) scores organized by subscales. We show scores for each testing round, separated by one refinement session.

Testers reported considerable variation in perceived workload, depending on the subscale. Mental demand and effort were consistently the highest and increased from round 1 to round 2 (Figure 10 [69]). Mental workload was highest, with a median score of 37.50 (IQR 21.25-57.50) for round 1 and a median score of 60.00 (IQR 56.3-63.8) for round 2. The median scores for total effort (a composite of mental and physical effort) were 42.50 (IQR 27.50-57.50) and 55.00 (IQR 46.3-63.8) for rounds 1 and 2, respectively.

### Competency Assessment

**Figure 11:**
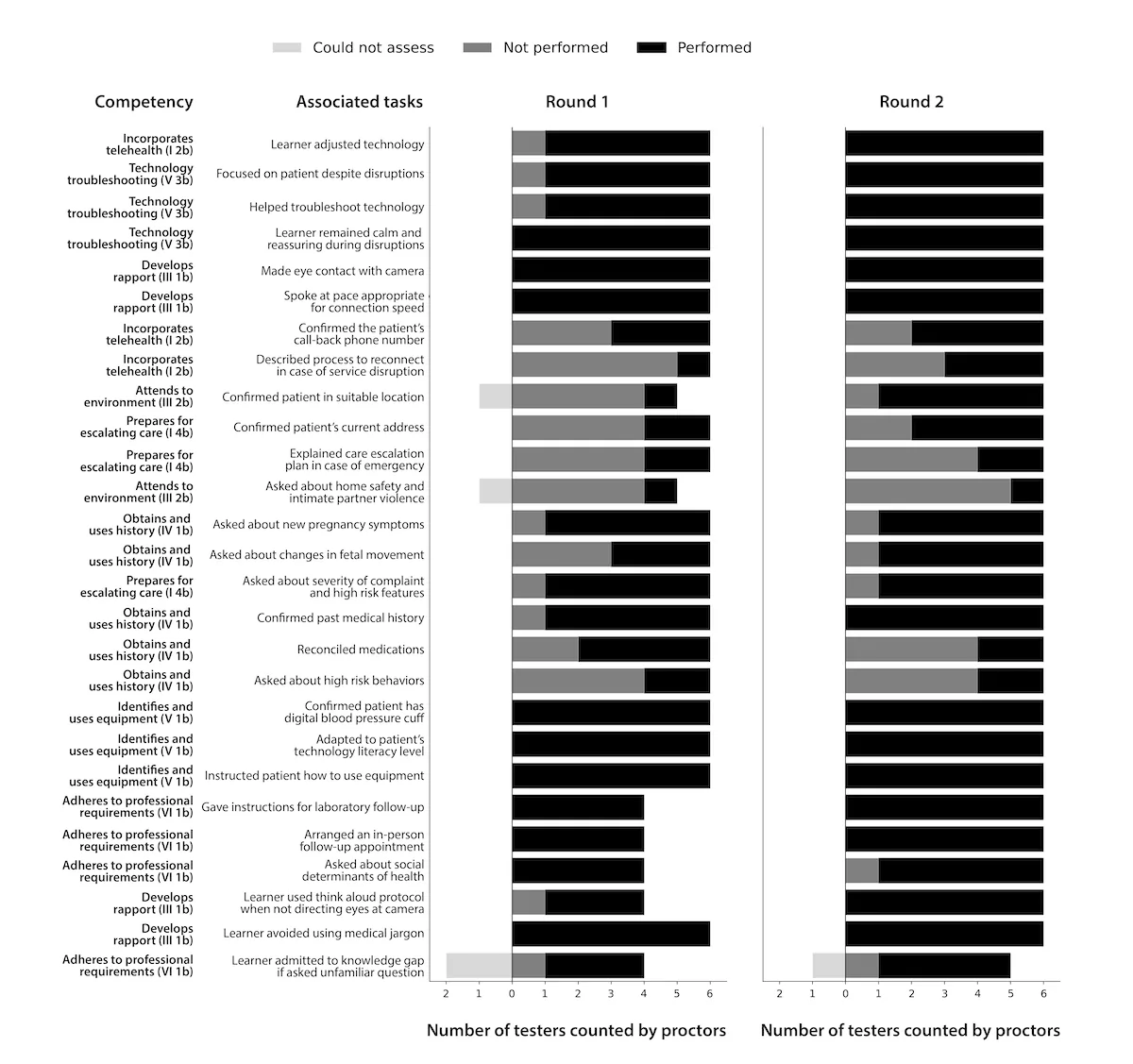
Individual task performance measured using a standardized rubric. Several tasks map to a single telehealth competency. We show task completion rates for each testing round, separated by one refinement session. Competencies were taken from the Association of American Medical Colleges (AAMC) white paper.

In the first round of testing, the proctor and observers could not score 3 tasks associated with 2 competencies (ie, “establishes therapeutic relationships and environments” and “supports solutions to ethical problems”). This problem was caused by ambiguities in the scoring guide’s language (Figure 11 [36]). Revising the language improved usability. One persistent problem was identifying when a learner fabricated information (eg, improvising the name of a regional laboratory) to preserve the illusion of the simulation. We therefore modified the SP script before piloting to discourage learner improvisation.

**Figure 12:**
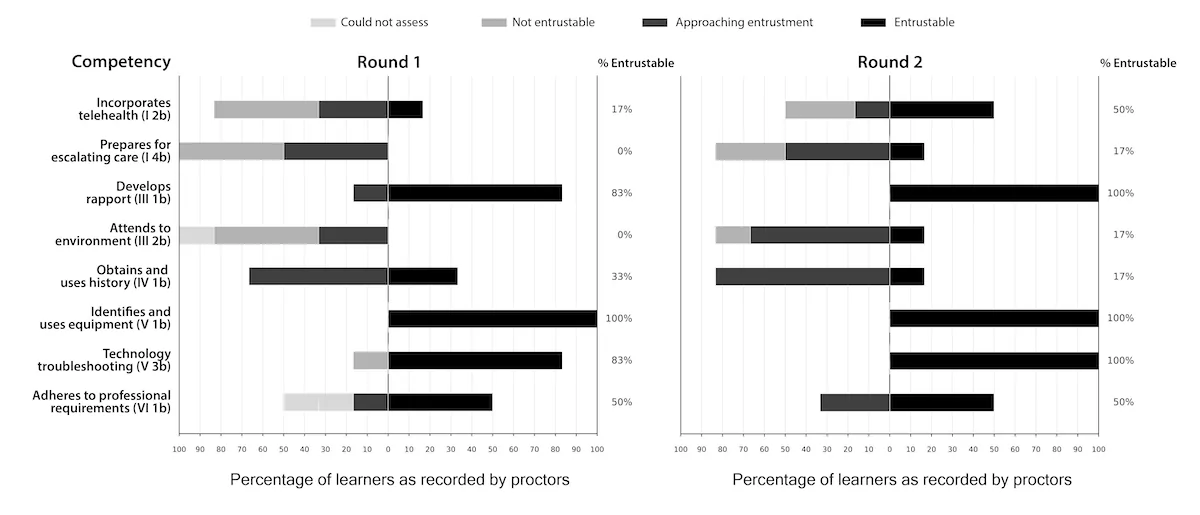
Telemedicine competencies measured using our scoring rubric. Each competency is a composite score of several related tasks. We calculated competencies only for testers with complete data. We show scores for each testing round, separated by one refinement session. Competencies were taken from the Association of American Medical Colleges (AAMC) white paper.

We summed proctor-scored task completion rates to calculate composite telemedicine competency scores (Figure 12 [36]). Testers tended to overlook telemedicine-specific tasks, such as gathering contact information, including the home address (AAMC competency I 2b); describing an emergency escalation plan (AAMC competency I 4b); or checking whether the patient was in a private setting and whether other household members were present (AAMC competency III 2b) [36].

## Discussion

### Overview

For this tutorial discussion, we first provide an abbreviated review of the most significant usability findings. We then offer a concept-level appraisal of our testing methods and insights for readers planning similar testing protocols. We conclude by comparing with the literature, highlighting gaps, unexplored research opportunities, and future directions.

### Principal Findings

Our usability evaluation offered valuable insights into the effectiveness of our educational simulation and opportunities for quality improvement. The data gathered using the ATA enabled the development team to track and communicate improvements to the research team and simulation staff. We identified 52 usability issues requiring prioritization and made 15 design revisions before piloting with learners. By the second round of testing, we removed critical chokepoints related to the audiovisual cross-check and added a mock EHR with synthetic patient data and fill-in data fields. The mock EHR provided the learner with visual cues that improved the telemedicine workflow and task completion rates. Our qualitative data clarified the root causes of usability issues and provided an efficient method for prioritizing revisions. We traced our biggest challenges to the fidelity of blood pressure data and the reliability of the evaluation rubric. We subsequently focused revision efforts on clarifying details in patient scripts, adding synthetic patient data—including a blood pressure flowsheet to the mock EHR—and modifying scoring guide language and definitions in the telemedicine rubric.

It was crucial to analyze the simulation in accordance with the workflow steps, expected tasks, and learner competencies. By measuring simulation steps and task performance independently, we could see when testers completed all scripted steps but overlooked clinical tasks. This helped distinguish undesirable technical distractors from scripted challenges. By removing usability issues that diverted testers’ attention from their tasks, testers could more effectively engage with the learning material. This also helped faculty more easily determine when simulation tasks were misaligned with learning objectives.

Testers successfully used the videoconferencing software, resolved technical issues, and established therapeutic rapport. By contrast, most testers neglected tasks necessary to optimize the telemedicine environment or prepare for a medical emergency. This is not unexpected; none of our testers received telemedicine training, whereas we gave a lecture to residents before the simulation.

For educators planning to include grading rubrics with their simulations, we recommend embedding scoring forms or debriefing guides into the testing instruments. In our example, we included the telemedicine competency rubric in the ATA. Our experience suggests a thorough appraisal of the evaluation materials by domain experts and participating faculty can improve construct validity by aligning simulation tasks and learning objectives with rubric terminology and instrument design. We also believe testing can improve instrument learnability and ease of use so that faculty can invest less time training proctors during piloting and implementation.

We used the SEQ, SUS, and NASA-TLX scores to compare our qualitative observations with usability benchmarks (ie, convergent validity). The range of SEQ scores (2 to 7) and SUS scores (65 to 100) helped identify the most challenging simulation components. The SEQ pinpointed issues in the synthetic data collected during the history and examination.

The NASA-TLX data helped estimate overall simulation difficulty. Responses indicated a relatively high score for mental workload compared with the other subscales (round 1: median score 37.50, IQR 21.25-57.50; round 2: median score 60.00, IQR 56.30-63.80). The scores may have increased due to the inclusion of a mock EHR. Considering these findings, we asked faculty whether the simulation should be modified. Obstetrics and family medicine faculty advised against changing the tasks because they aligned with the educational goals and learning objectives. During piloting (illustrative vignette in Textbox 2; key tutorial insights in Textbox 3), residents were indeed able to complete the simulation but struggled with several tasks, including developing a preemptive care escalation plan and instructing SPs on the use of a digital blood pressure cuff. This suggests that our clinical scenario and materials provided sufficient scaffolding for learners to engage effectively with the material while gently challenging them with unfamiliar tasks.

**Key tutorial insights.**

**Key tutorial insights**

- Agile Task Analysis combined with simulations using a “think aloud” protocol offers a practical strategy for identifying critical design issues.
- It is crucial to include instructional materials, scoring guides, and debriefing instruments in usability data collection instruments. This practice can help disambiguate simulation design issues from learner competency gaps.
- The System Usability Scale is a practical method for quickly benchmarking the overall perceived usability of an educational simulation.
- The addition of the Single Ease Question can help pinpoint the source of usability issues when global usability assessments raise concerns.
- The NASA (National Aeronautics and Space Administration) Task Load Index provides a “quick and dirty” method to estimate the difficulty of an educational simulation and guide future calibration.

### Application of Findings to Simulation Development

This tutorial advances simulation science in several ways. First, we demonstrated how to leverage usability methods used in technology evaluations to systematically assess educational simulations before piloting with learners. Validated, theory-driven instruments can provide insights into simulation effectiveness, ease of use, and user satisfaction [22,82-84]. These methods are technology-agnostic and, with little modification, are suitable for evaluating simulation constructs such as engagement, cognitive load, skill acquisition, and competence.

While this study offers turnkey methods for formative usability testing, the choice of any single instrument should be driven by the learning objectives, the simulation’s attributes, and the phase of the development life cycle. Not all methods are necessary for every simulation study. Researchers should add or remove components in a modular fashion. For example, user satisfaction with simulation data may be incidental to the educational program objectives; studies show self-assessment is an unreliable estimate of teaching effectiveness [85,86]. We included the SSES in our protocol because we believed fellow educators could provide useful appraisals of our simulation and its educational value.

Second, our approach addressed design challenges unique to educational simulations [22,23,74,87]. We concurrently evaluated simulation design features and learner competencies to align teaching modalities with educational objectives and to balance ecological validity with simulation complexity [4,11,12,20,88,89]. Our instruments were well suited to observing technology use and should be considered for technology-forward educational initiatives that include EHRs, mobile devices, artificial intelligence, and computerized decision support. Unlike traditional usability testing, our goal was not to remove opportunities for user error [17,21,73,80,90-92]. Educational simulations must enable learning through experimentation, discovery, and even failure [4,42,93,94]. We therefore used the ATA to record whether testers had the opportunity to demonstrate their skills during the simulation; the simulation could proceed as intended even if a tester failed a task.

Third, our methods can be used early in the product development life cycle with an array of testers [2,4,9]. While best-practice simulation guidelines recommend piloting simulations with learners, there are practical challenges [2]. Recruiting enough target learners can be impractical. Moreover, testing and piloting serve different but equally important roles. Because simulations can be challenging or stressful for learners, pilots may not be the ideal means of measuring usability dimensions such as learnability, error tolerance, and satisfaction [95-98]. Testing with experts can assess the fidelity of the case, the accuracy of the synthetic data, the appropriateness of the learning objectives, and the difficulty of tasks [41-43,99].

Fourth, we believe our methods are flexible, cost-effective, and scalable. We used low-cost tools that could be adapted to resource-constrained settings, such as smaller academic centers and schools in low- and middle-income countries [73,74,100]. Many of the instruments described herein, including the SUS, NASA-TLX, and SSES, have been validated in languages such as Chinese, Korean, Spanish, French, and Croatian [69,101-107]. These methods do not require large numbers of testers. We recommend multiple short testing rounds with as few as 5 testers; this provides sufficient data to guide refinements [108].

### Comparison With Prior Work

With the expansion of advanced simulation modalities, such as AR, VR, and AI, software developers are increasingly recognizing the role of usability testing in the product development life cycle [109-114]. There are already protocols for a range of tools, including AR, VR, digital twins, and serious games [8,14,15]. While most reports focus on the technology, several have evaluated the broader simulation context. Anton and colleagues described an evaluation of 2 emergency medicine simulations that combined learner eye tracking and faculty ratings on the Non-Technical Skills for Surgeons Scale [14]. Silva et al [15] applied a more comprehensive approach by evaluating a simulation manikin for teaching cardiopulmonary resuscitation. They created a data collection instrument based on a basic life support guide and scored learners using a 16-item Likert-type scale. The researchers recorded time on task and administered the SUS after each encounter. In this manner, they operationalized usability in terms of the dimensions of effectiveness, efficiency, and satisfaction. Together, these reports provide complementary perspectives on the evaluation of simulation-based instructional modules: Silva et al [15] focused on the physical usability of manikins, whereas Anton et al [14] focused on clinicians’ cognitive skills.

Da Silva and Dubrowski [8] described one of the first attempts to incorporate usability methods early in the development life cycle. They conducted a series of design sprints to evaluate a conflict-management scenario between a nursing student and a senior nurse. The team tested 2 scenarios and recruited 20 nursing students. After each simulation, the team conducted exit interviews and administered the Simulation Design Scale (SDS)—a validated 20-item instrument measuring the quality and effectiveness of simulation-based learning experiences [115]. Unlike the SSES, which focuses on learners’ satisfaction with the instructional approach, the SDS focuses on missing or underdeveloped simulation elements. Da Silva and Dubrowski [8] used the SDS as a quality metric and tracked improvement in scores across iterations. We believe this work is an important milestone toward developing a metrics-driven usability strategy.

### Strengths, Limitations, and Next Steps to Improve Testing Protocols

We believe the application of usability methods to simulation-based education remains underexplored [41,42,99]. To our knowledge, our report and related preliminary work provide the most complete descriptions of mixed methods usability testing for educational simulations. Combining subjective measures (eg, user satisfaction and ease of use) with objective performance data (eg, task completion rates, error tolerance) can maximize insights into technical functionality and educational value [57,116,117].

These observations notwithstanding, several limitations may affect the generalizability of our work. We identified new usability issues after 2 refinement sessions, suggesting we had not reached saturation. Unfortunately, our team faced time and resource constraints; the number of iterations was, in part, dependent on curriculum deadlines and the number of available testers. To our knowledge, there is no universally accepted number of testers or testing rounds that guarantees researchers will find all usability issues. The usability literature, however, describes an issue-discovery curve with diminishing returns; as few as 5 testers may identify between 80% and 90% of critical issues [17]. Experts therefore recommend conducting several small testing cycles with 5 users at a time [17,118-121].

This report describes a single-site, single-SP, quality-improvement design. This raises several validity issues that usability specialists must consider. We conducted all usability testing with a single SP to control for simulation variability. However, our SP’s familiarity with the case may have limited our ability to identify weak prompts, responses, or missing information. In the future, incorporating multiple SPs could reveal variations in script interpretation and behavior. We also observed variation in practice behaviors among subject-matter experts as a function of experience, specialty, and training. This suggests that institutions may inadvertently incorporate provincial practice patterns and local health system norms into their simulation designs. This could create standardization challenges, since usability studies tend to compare testers' performance against an established workflow or an approved set of product design requirements. In our example, we compared usability findings to local workflow and learning objectives. Researchers designing future studies may elect to use an external reference standard, such as a prerecorded best-practice encounter developed by a professional society.

We did not conduct interrater reliability tests for our rubric. In general, calculating agreement statistics falls outside the scope of usability testing during the product design phase. Instead, several members of the research team independently coded each task. We then compared scores and resolved discrepancies through discussion. Task descriptions with vague or ambiguous language were updated during the refinement phases. We believe the next step in our research program is to measure the interrater reliability of our rubric by asking faculty to independently rate learners who have completed the simulation and calculating an agreement statistic (eg, Cohen κ or Fleiss κ) [122].

We qualitatively coded all usability findings using the usability heuristics for health information technology by Zhang et al [80]. While useful for categorization, the codes did not indicate priority or provide insights for remediation. We believe there are opportunities to develop and validate usability heuristics specific to educational simulations. Ideally, codes informed by educational theory would provide design guidance to educators.

Finally, we see future opportunities to test additional usability measurement tools, including the SDS, heuristic inspections, and cognitive walkthroughs [17,22,115]. This could help enrich our logic models that connect usability tools to evaluation dimensions, such as realism, memorability, completeness, and predicted future learner performance. While videoconferencing may not be required for most simulations, other teams might use this capability to link distributed resources, such as testers, researchers, SPs, and software. To support these kinds of remote testing initiatives, researchers may distribute questionnaires digitally and trial wearable technologies, such as eye-tracking lenses and wearable activity trackers, including wristbands and smartwatches.

### Conclusions

We believe this tutorial illustrates a practical, reproducible, and resource-sensitive method for usability testing educational simulations before piloting with target learners. While this report describes a single-site, single-SP, quality-improvement design, we strove to include published, validated instruments with claims of generalizability and relatively low-cost testing methods to permit replication or context-appropriate adaptation. We have included appendices with our instruments and encourage readers to adapt and apply these methods to a range of simulations. While we believe this monograph provides simulationists with a turnkey toolkit to conduct similar assessments, further studies using psychometrically validated instruments are needed to clarify the best educational use cases, evaluate additional constructs relevant to teaching and learning, and, when relevant, incorporate additional tests to measure the interrater reliability of learner assessment instruments.

## Appendix

Multimedia Appendix 1 Demographic questionnaire.

Multimedia Appendix 2 Agile task analysis with embedded telemedicine competency rubric.

Multimedia Appendix 3 List of Single Ease Questions (SEQ) for sets of related steps.

Multimedia Appendix 4 Adapted post-simulation System Usability Scale (SUS) and Satisfaction with Simulation Experience Survey (SSES).

Multimedia Appendix 5 Unweighted NASA Task Load Index (NASA-TLX).

Multimedia Appendix 6 Semistructured simulation debrief guide.

Multimedia Appendix 7 Structural codes data dictionary for usability issue coding.

Multimedia Appendix 8 Usability issue log template with sample entries.

Multimedia Appendix 9 Telehealth competency rubric used during resident piloting.

Multimedia Appendix 10 Printed telemedicine memory aid.

## Abbreviations

AAMC: Association of American Medical Colleges
AR: augmented reality
ATA: Agile Task Analysis
EHR: electronic health record
FCM: family and community medicine
HTA: hierarchical task analysis
NASA: National Aeronautics and Space Administration
NASA-TLX: NASA Task Load Index
OB/GYN: obstetrics and gynecology
OUSCM: University of Oklahoma School of Community Medicine
SDS: Simulation Design Scale
SEQ: Single Ease Question
SP: standardized patient
SSES: Satisfaction With Simulation Experience Scale
SUS: System Usability Scale
VR: virtual reality

## Data Availability

The datasets generated or analyzed during this study are available from the corresponding author on reasonable request.
