# Supplementary material for: How to Conduct Usability Testing of Medical Education Simulations: Methods Tutorial Using a Telemedicine Simulation for Maternal Care
Source: JMIR Med Educ. 2026 Sep 9;12:e102697. doi: 10.2196/102697 (PMC13601883; doi:10.2196/102697)
Supplement: Supplementary file 1 [file mededu_v12i1e102697_app1.docx]

Appendix 1: Demographic Questionnaire

| **Demographics** | 1. Are you a faculty member, resident, or student? | | | |  | | |
| --- | --- | --- | --- | --- | --- | --- | --- |
|  | 2. What is your age? | | | |  | | |
|  | 3. What is your level of training? | | | |  | | |
|  | 4. How many years have you practiced your specialty, including residency?   (for post-graduates only) | | | |  | | |
|  | 5. What specialty are you in or considering? | | | |  | | |
| **Technology Literacy** | 6. Do you use a computer at least once a week? | | | | Yes | | No |
|  | 7. Do you use a smartphone, mobile device, or other portable technology? | | | | Yes | | No |
|  | 8. Have you used Zoom or similar software for videoconferencing? | | | | Yes | | No |
| **Perceptions & Attitudes** | 9. Have you ever observed a telehealth encounter? | | | | Yes | | No |
|  | 10. Have you ever participated in a telehealth encounter as a provider? | | | | Yes | | No |
|  | 11. Have you ever participated in a telehealth encounter as a patient? | | | | Yes | | No |
|  | 12. Have you ever received any formal instruction in telemedicine? | | | | Yes | | No |
|  | 13. I have a good understanding of how telemedicine is practiced (e.g., knowledge of potential uses, technology, and workflow). | | | | | | |
|  | Strongly disagree | Disagree | Neutral | Agree | | Strongly agree | |
|  | 14. I am familiar with the types of technology used in telemedicine. | | | | | | |
|  | Strongly disagree | Disagree | Neutral | Agree | | Strongly agree | |
|  | 15. I think telemedicine is a good option for care even when face-to-face is available. | | | | | | |
|  | Strongly disagree | Disagree | Neutral | Agree | | Strongly agree | |
|  | 16. I will likely use telemedicine in my practice | | | | | | |
|  | Strongly disagree | Disagree | Neutral | Agree | | Strongly agree | |
