# Supplementary material for: How to Conduct Usability Testing of Medical Education Simulations: Methods Tutorial Using a Telemedicine Simulation for Maternal Care
Source: JMIR Med Educ. 2026 Sep 9;12:e102697. doi: 10.2196/102697 (PMC13601883; doi:10.2196/102697)
Supplement: Supplementary file 3 [file mededu_v12i1e102697_app2.docx]

## Appendix 2: Agile Task Analysis with Embedded Telemedicine Competency Rubric

*Note: The residency instructors used the competency rubric without the ATA components during piloting with residents. The final competency rubric instrument is included in a separate appendix.*
