# Supplementary material for: How to Conduct Usability Testing of Medical Education Simulations: Methods Tutorial Using a Telemedicine Simulation for Maternal Care
Source: JMIR Med Educ. 2026 Sep 9;12:e102697. doi: 10.2196/102697 (PMC13601883; doi:10.2196/102697)
Supplement: Supplementary file 4 [file mededu_v12i1e102697_app3.docx]

## Appendix 3: List of Single Ease Questions (SEQ) for Sets of Related Steps

**Overall, how difficult or easy did you find the task of interpreting the materials?**

| **Very difficult** |  |  |  |  |  | **Very Easy** |
| --- | --- | --- | --- | --- | --- | --- |
| **1** | **2** | **3** | **4** | **5** | **6** | **7** |
| □ | □ | □ | □ | □ | □ | □ |

**Overall, how difficult or easy did you find this task of starting a telemedicine encounter?**

| **Very difficult** |  |  |  |  |  | **Very Easy** |
| --- | --- | --- | --- | --- | --- | --- |
| **1** | **2** | **3** | **4** | **5** | **6** | **7** |
| □ | □ | □ | □ | □ | □ | □ |

**Overall, how difficult or easy did you find completing telemedicine workflow tasks?**

| **Very difficult** |  |  |  |  |  | **Very Easy** |
| --- | --- | --- | --- | --- | --- | --- |
| **1** | **2** | **3** | **4** | **5** | **6** | **7** |
| □ | □ | □ | □ | □ | □ | □ |

**Overall, how difficult or easy did you find the task of history taking?**

| **Very difficult** |  |  |  |  |  | **Very Easy** |
| --- | --- | --- | --- | --- | --- | --- |
| **1** | **2** | **3** | **4** | **5** | **6** | **7** |
| □ | □ | □ | □ | □ | □ | □ |

**Overall, how difficult or easy did you find the task of gathering vital signs?**

| **Very difficult** |  |  |  |  |  | **Very Easy** |
| --- | --- | --- | --- | --- | --- | --- |
| **1** | **2** | **3** | **4** | **5** | **6** | **7** |
| □ | □ | □ | □ | □ | □ | □ |

**Overall, how difficult or easy did you find the task of communicating a diagnosis and plan?**

| **Very difficult** |  |  |  |  |  | **Very Easy** |
| --- | --- | --- | --- | --- | --- | --- |
| **1** | **2** | **3** | **4** | **5** | **6** | **7** |
| □ | □ | □ | □ | □ | □ | □ |
