# Supplementary material for: How to Conduct Usability Testing of Medical Education Simulations: Methods Tutorial Using a Telemedicine Simulation for Maternal Care
Source: JMIR Med Educ. 2026 Sep 9;12:e102697. doi: 10.2196/102697 (PMC13601883; doi:10.2196/102697)
Supplement: Supplementary file 5 [file mededu_v12i1e102697_app4.docx]

## Appendix 4: Adapted Post-Simulation System Usability Scale (SUS) and Satisfaction with Simulation Experience Survey (SSES)

|  | P**LEASE CIRCLE THE CHOICE THAT BEST REFLECTS YOUR BELIEF, PERSPECTIVE, OR EXPERIENCE** | | | | | | | |
| --- | --- | --- | --- | --- | --- | --- | --- | --- |
|  |  |  |  |  |  |  |  |  |
| 1 | I thought the instructions for the simulation were clear* |  | Strongly disagree | Disagree | Neutral | Agree | Strongly agree |  |
| 2 | I would need to prepare a lot to participate in a similar simulation* |  | Strongly disagree | Disagree | Neutral | Agree | Strongly agree | RS |
| 3 | I did not think the simulated scenario flowed smoothly* |  | Strongly disagree | Disagree | Neutral | Agree | Strongly agree | RS |
| 4 | I thought the simulation was easy to complete* |  | Strongly disagree | Disagree | Neutral | Agree | Strongly agree |  |
| 5 | I felt confident completing the required steps of the simulation* |  | Strongly disagree | Disagree | Neutral | Agree | Strongly agree |  |
| 6 | I thought this simulation was unnecessarily complex* |  | Strongly disagree | Disagree | Neutral | Agree | Strongly agree | RS |
| 7 | I think I would need a lot of technical support to participate in a similar simulation* |  | Strongly disagree | Disagree | Neutral | Agree | Strongly agree | RS |
| 8 | I did not think the simulation was representative of a real-life experience* |  | Strongly disagree | Disagree | Neutral | Agree | Strongly agree | RS |
| 9 | I would participate in similar simulations to help me learn more about telemedicine* |  | Strongly disagree | Disagree | Neutral | Agree | Strongly agree |  |
| 10 | I imagine most learners would be able to participate in this simulation* |  | Strongly disagree | Disagree | Neutral | Agree | Strongly agree |  |
| 11 | I received feedback during the debriefing that helped me learn^†^ |  | Strongly disagree | Disagree | Neutral | Agree | Strongly agree |  |
| 12 | The simulation developed my telemedicine skills^†^ |  | Strongly disagree | Disagree | Neutral | Agree | Strongly agree |  |
| 13 | The simulation developed my clinical decision-making ability^†^ |  | Strongly disagree | Disagree | Neutral | Agree | Strongly agree |  |
| 14 | The simulation tested my clinical reasoning skills^†^ |  | Strongly disagree | Disagree | Neutral | Agree | Strongly agree |  |
| 15 | The simulation tested my clinical ability^†^ |  | Strongly disagree | Disagree | Neutral | Agree | Strongly agree |  |
| 16 | The simulation helped me to learn to manage technical challenges^†^ |  | Strongly disagree | Disagree | Neutral | Agree | Strongly agree |  |
| 17 | The simulation was a valuable learning experience^†^ |  | Strongly disagree | Disagree | Neutral | Agree | Strongly agree |  |
| 18 | The simulation caused me to reflect on my telemedicine skills^†^ |  | Strongly disagree | Disagree | Neutral | Agree | Strongly agree |  |
| 19 | The simulation helped me identify my strengths and growth opportunities^†^ |  | Strongly disagree | Disagree | Neutral | Agree | Strongly agree |  |
| 20 | Discussing the simulation enhanced my learning^†^ |  | Strongly disagree | Disagree | Neutral | Agree | Strongly agree |  |
| * System Usability Scale ^†^ Satisfaction with Simulation Experience Scale RS: Reverse scored | | | | | | | | |
