# Supplementary material for: How to Conduct Usability Testing of Medical Education Simulations: Methods Tutorial Using a Telemedicine Simulation for Maternal Care
Source: JMIR Med Educ. 2026 Sep 9;12:e102697. doi: 10.2196/102697 (PMC13601883; doi:10.2196/102697)
Supplement: Supplementary file 6 [file mededu_v12i1e102697_app5.docx]

## Appendix 5: Unweighted NASA Task Load Index (NASA-TLX)

|  | **PLEASE DRAW AN “X” ON THE SCALE TO BEST INDICATE YOUR EXPERIENCE DURING THE SIMUATION** | | | | | | |  |
| --- | --- | --- | --- | --- | --- | --- | --- | --- |
| **1** | How mentally demanding was the simulation? |  | Very low |  |  |  | Very high |  |
| **2** | How physically demanding was the simulation? |  | Very low |  |  |  | Very high |  |
| **3** | How hurried or rushed was the pace of the simulation? |  | Very low |  |  |  | Very high |  |
| **4** | How successful were you in accomplishing the tasks of the simulation? |  | Perfect |  |  |  | Failure |  |
| **5** | How hard did you have to work to accomplish your level of performance? |  | Very low |  |  |  | Very high |  |
| **6** | How insecure, discouraged, irritated, and stressed were you during the simulation? |  | Very low |  |  |  | Very high |  |
