# Supplementary material for: How to Conduct Usability Testing of Medical Education Simulations: Methods Tutorial Using a Telemedicine Simulation for Maternal Care
Source: JMIR Med Educ. 2026 Sep 9;12:e102697. doi: 10.2196/102697 (PMC13601883; doi:10.2196/102697)
Supplement: Supplementary file 7 [file mededu_v12i1e102697_app6.docx]

## Appendix 6: Semi-Structured Simulation Debrief Guide

1. **“Overall, how easy did you find the task of completing this telemedicine encounter?”**

| **Very difficult** |  |  |  |  |  | **Very Easy** |
| --- | --- | --- | --- | --- | --- | --- |
| **1** | **2** | **3** | **4** | **5** | **6** | **7** |
| □ | □ | □ | □ | □ | □ | □ |

2. **“Did you have any problems just now completing the case? If so, please describe each problem.”**
3. **What did you think were the most valuable learning aspects of the simulation? Why?”**
