# Supplementary material for: How to Conduct Usability Testing of Medical Education Simulations: Methods Tutorial Using a Telemedicine Simulation for Maternal Care
Source: JMIR Med Educ. 2026 Sep 9;12:e102697. doi: 10.2196/102697 (PMC13601883; doi:10.2196/102697)
Supplement: Supplementary file 8 [file mededu_v12i1e102697_app7.docx]

## Appendix 8: Structural Codes Data Dictionary for Usability Issue Coding

Adapted from Zhang et al, J Biomedical Informatics, 2003

| **Name** | **Description** |
| --- | --- |
| **Consistency** | *Consistency and standards.* Learners and proctors should not have to wonder whether different words, situations, or actions mean the same thing. Standards and conventions in simulation and clinical care should be followed. |
| **Visibility** | *Visibility of simulation state.* Learners and proctors should be informed about what is going on throughout the simulation through appropriate patient responses and display of information. |
| **Match** | *Match between simulation and world.* The data and experience of the simulation perceived by learners should match the clinical systems and disease processes in the real world. |
| **Minimalist** | *Minimalist.* Any extraneous information for the learners or proctors is a distraction and obscures the learning objectives or observations. |
| **Memory** | *Minimize memory load.* Learners or proctors should not be required to memorize a lot of information commonly available to carry out tasks. Memory load reduces capacity to carry out the main tasks. |
| **Feedback** | *Informative feedback.* The system, simulation, and patient should give prompt and informative responses to learners. |
| **Flexibility** | *Flexibility and efficiency.* Learners are always different. Give learners the flexibility of adapting or improvising to accelerate their learning and performance. |
| **Message** | *Good error messages.* The language and communication should be informative enough such that learners can understand the nature of errors and learn from errors. |
| **Error** | *Prevent errors.* It is always better to design simulations that prevent errors from happening in the first place. Simulations should not include artificial circumstances that promote or propagate learner errors. |
| **Closure** | *Clear closure.* Every task has a beginning and an end. Learners should be clearly notified about the completion of a task. |
| **Undo** | *Reversible actions.* Learners should be able to recover from errors when recognized during simulation. Reversible actions encourage exploratory learning. |
| **Language** | *Use users’ language.* The language should be always presented in a form understandable by the intended learners and proctors. |
| **Control** | *Users in control.* Do not give learners the impression that they are controlled by the simulation and cannot clinically adapt or improvise. |
| **Document** | *Help and documentation.* Always provide help if it supports the learning process or reinforces the learning objectives. |
| **Wildcard** | *Wildcard.* Used for errors that do not fit other categories. |
