# Supplementary material for: How to Conduct Usability Testing of Medical Education Simulations: Methods Tutorial Using a Telemedicine Simulation for Maternal Care
Source: JMIR Med Educ. 2026 Sep 9;12:e102697. doi: 10.2196/102697 (PMC13601883; doi:10.2196/102697)
Supplement: Supplementary file 9 [file mededu_v12i1e102697_app8.docx]

## Appendix 9: Usability Issue Log Template with Sample Entries

| # | **Obs** | **Finding** | **Code(s)** | **Valence** | **Priority** | **Fixed?** | **Intervention** |
| --- | --- | --- | --- | --- | --- | --- | --- |
| 7 | CL | The tester expected to see a flowsheet of blood pressure values in the mock electronic record | Match | Negative | High | Yes | Added a flowsheet with additional blood pressure values in LearningSpace software |
| 8 | BL | Need to include photographs of physical findings such as leg edema | Feedback | Negative | High | Yes | Gave SP printed photos of physical exam findings sourced from licensed photo stock |
| 8 | MA | Setting up dual windows for Zoom and LearningSpace added technical setup challenges | Visibility | Negative | Low | No | None; this will be managed by the Simulation Center staff |
|  |  |  |  |  |  |  |  |
|  |  |  |  |  |  |  |  |
|  |  |  |  |  |  |  |  |
|  |  |  |  |  |  |  |  |
|  |  |  |  |  |  |  |  |

#: Usability finding	Obs: Researcher initials	Code: Structural qualitative code	Valence: Positive or negative
