# Supplementary material for: How to Conduct Usability Testing of Medical Education Simulations: Methods Tutorial Using a Telemedicine Simulation for Maternal Care
Source: JMIR Med Educ. 2026 Sep 9;12:e102697. doi: 10.2196/102697 (PMC13601883; doi:10.2196/102697)
Supplement: Supplementary file 10 [file mededu_v12i1e102697_app9.docx]

## Appendix 7: Telehealth Competency Rubric Used During Resident Piloting

Learner name: ________________________Training Level: **MS III	MS IV	PA II	PGY 1	PGY 2	PGY 3	PGY4**

*Circle one*

Program: **Obstetrics and Gynecology	Family Medicine	Internal Medicine**

*Circle one*

Evaluator: ________________________________________________ Date: ______________________

**Telemedicine Competencies Assessment Instructions**

For each task, please indicate whether you observed the learner complete the task by indicating “not observed” or “observed.” In some simulations, a task may not be applicable. Very few learners – including experienced faculty – will complete all the tasks. This is particularly true earlier in training.

For each competency, circle or check only one category that best describes the level of proficiency. **Few learners will be entrustable in every category.**

| **DOMAIN III**  **COMPETENCY 2b** | | **COMMUNICATION VIA TELEHEALTH** | | | |
| --- | --- | --- | --- | --- | --- |
| *Establishes therapeutic relationships and environments during video visits, such as attending to disruptions related to privacy, lighting, sound, and attire. This includes optimizing teleconferencing settings and using environmental cues and resources to gain insight into the patient’s health status.* | | | | | |
| **Item** | **Description of behavior or activity** | | **Assessment** | | **Comments**  **(optional)** |
| **1** | The learner completed a **technology “cross-check”** to confirm that the patient could hear and see him/her/them | | □  Not observed | □  Observed |  |
| **2** | The learner confirmed the patient was in a **suitable private location** (for example, not driving or in a public setting) | | □  Not observed | □  Observed |  |

| **COMPETENCY SCORING** | |
| --- | --- |
| **Write in the number of the number of tasks observed complete** | **Total** |
| **Scoring: Not yet entrustable 0 \| Approaching entrustment 1 \| Entrustable 2** |  |

| **DOMAIN I**  **COMPETENCY 2b** | | **PATIENT SAFETY & APPROPRIATE USE OF TELEHEALTH** | | | |
| --- | --- | --- | --- | --- | --- |
| *Works with diverse patients and caregivers to evaluate and remedy patient and practice barriers to incorporating telehealth into patient care (e.g., access to and comfort with technology)* | | | | | |
| **Item** | **Description of behavior or activity** | | **Assessment** | | **Comments**  **(optional)** |
| **1** | The learner confirmed the **patient’s contact phone number** in case of a disruption of service | | □  Not observed | □  Observed |  |
| **2** | The learner **described their process to re-establish contact** if there was a disruption in service (*e.g.*, software crash, internet failure) | | □  Not observed | □  Observed |  |

| **COMPETENCY SCORING** | |
| --- | --- |
| **Write in the number of the number of tasks observed complete** | **Total** |
| **Scoring: Not yet entrustable 0** \| **Approaching entrustment 1** \| **Entrustable 2** |  |

| **DOMAIN I**  **COMPETENCY 4b** | | **PATIENT SAFETY & APPROPRIATE USE OF TELEHEALTH** | | | |
| --- | --- | --- | --- | --- | --- |
| *Prepares for and escalates care when patient safety is at risk during a telehealth encounter. This includes confirming the patient’s location.* | | | | | |
| **Item** | **Description of behavior or activity** | | **Assessment** | | **Comments**  **(optional)** |
| **1** | The learner confirmed the patient’s **geographic location and address** in the event of an emergency | | □  Not observed | □  Observed |  |
| **2** | The learner explained a **plan to escalate care** if the patient had an emergency (*e.g.*, calling emergency services) | | □  Not observed | □  Observed |  |
| **3** | The learner tried to establish the **severity of new complaints**, asking about symptoms suggestive of preeclampsia, including shortness of breath, new or worsening headaches, visual changes, epigastric or right upper quadrant pain, or excessive swelling, particularly of the hands and face | | □  Not observed | □  Observed |  |

| **COMPETENCY SCORING** | |
| --- | --- |
| Write in the number of the number of tasks observed complete | **Total** |
| **Scoring: Not yet entrustable 0 – 1** \| **Approaching entrustment 2** \| **Entrustable 3** |  |

| **DOMAIN III**  **COMPETENCY 1b** | | **COMMUNICATION VIA TELEHEALTH** | | | |
| --- | --- | --- | --- | --- | --- |
| *Develops an effective rapport with patients via video visits, attending to eye contact, tone, body language, and nonverbal cues. This includes using a universal communication precautions approach and “Think-Aloud” protocol.* | | | | | |
| **Item** | **Description of behavior or activity** | | **Assessment** | | **Comments**  **(optional)** |
| **1** | The learner made regular **eye contact with the camera** | | □  Not observed | □  Observed |  |
| **2** | The learner **spoke at an adjusted (*e.g.*, slower) pace to accommodate** the video teleconferencing connection speed | | □  Not observed | □  Observed |  |
| **3** | The learner used a **“Think Aloud” protocol** to describe what he/she/they were doing when not looking at the patient | | □  Not observed | □  Observed |  |
| **4** | The learner **avoided using medical jargon** | | □  Not observed | □  Observed |  |

| **COMPETENCY SCORING** | |
| --- | --- |
| **Write in the number of the number of tasks observed complete** | **Total** |
| **Scoring: Not yet entrustable 0 – 1 \| Approaching entrustment 2 – 3 \| Entrustable 4** |  |

| **DOMAIN IV**  **COMPETENCY 1b** | | **DATA COLLECTION AND ASSESSMENT VIA TELEHEALTH** | | | |
| --- | --- | --- | --- | --- | --- |
| *Obtains history during a real or simulated encounter and incorporates the information into a differential diagnosis and management plan. This includes reviewing prescription medications, over-the-counter medications, nutraceuticals, and supplements.* | | | | | |
| **Item** | **Description of behavior or activity** | | **Assessment** | | **Comments**  **(optional)** |
| **1** | The learner asked if the patient had any **new symptoms** associated with pregnancy, such as nausea or depressed mood | | □  Not observed | □  Observed |  |
| **2** | The learner asked about any changes or decreases in **fetal movement, vaginal bleeding, or loss of fluid**. | | □  Not observed | □  Observed |  |
| **3** | The learner reviewed the patient’s past medical history, asking about **any known history of hypertension or prior elevated pressures** | | □  Not observed | □  Observed |  |
| **4** | The learner **reviewed medications** by asking about adherence with prescriptions ***or*** if there were new over-the-counters and supplements | | □  Not observed | □  Observed |  |

| **COMPETENCY SCORING** | |
| --- | --- |
| **Write in the number of the number of tasks observed complete** | **Total** |
| **Scoring: Not yet entrustable 0 – 1 \| Approaching entrustment 2 \| Entrustable 3 – 4** |  |

| **DOMAIN V**  **COMPETENCY 1b** | | **TECHNOLOGY FOR TELEHEALTH** | | | |
| --- | --- | --- | --- | --- | --- |
| *Identifies and can use the equipment needed for the intended service at both originating and distant sites.* | | | | | |
| **Item** | **Description of behavior or activity** | | **Assessment** | | **Comments**  **(optional)** |
| **1** | The learner asked the patient **if they have home equipment**, such as a blood pressure cuff | | □  Not observed | □  Observed |  |
| **2** | The learner adapted to the **patient’s technology literacy** by asking about their comfort level and helping without using **technical jargon** | | □  Not observed | □  Observed |  |
| **3** | The learner confirmed that patient has been trained and is comfortable taking their **home pressures** (*e.g.*, the patient has feet on floor, back and arm supported, not talking, empty bladder) | | □  Not observed | □  Observed |  |

| **COMPETENCY SCORING** | |
| --- | --- |
| **Write in the number of the number of tasks observed complete** | **Total** |
| **Scoring: Not yet entrustable 0 – 1 \| Approaching entrustment 2 \| Entrustable 3** |  |

| **DOMAIN V**  **COMPETENCY 3b** | | **TECHNOLOGY FOR TELEHEALTH** | | | |
| --- | --- | --- | --- | --- | --- |
| *Demonstrates how to troubleshoot technology failures and optimize settings with the technology being used.* | | | | | |
| **Item** | **Description of behavior or activity** | | **Assessment** | | **Comments**  **(optional)** |
| **1** | The learner stayed **focused upon the patient** despite technology challenges or disruptions | | □  Not observed | □  Observed |  |
| **2** | The learner helped the patient **troubleshoot basic technology failures** | | □  Not observed | □  Observed |  |
| **3** | The learner **remained calm, reassuring, and composed** when the patient encountered difficulties | | □  Not observed | □  Observed |  |

| **COMPETENCY SCORING** | |
| --- | --- |
| **Write in the number of the number of tasks observed complete** | **Score** |
| **Scoring: Not yet entrustable 0 – 1 \| Approaching entrustment 2 \| Entrustable 3** |  |

| **DOMAIN VI**  **COMPETENCY 3b** | | **ETHICAL PRACTICES & LEGAL REQUIREMENTS FOR TELEHEALTH** | | | |
| --- | --- | --- | --- | --- | --- |
| *Identifies and supports solutions that mitigate ethical problems and adhere to professional requirements in telehealth.* | | | | | |
| **Item** | **Description of behavior, task, or activity** | | **Score** | | **Comments (optional)** |
| **1** | The learner communicated a suspected **diagnosis of gestational hypertension** to the patient and offered a differential | | □  Not observed | □  Observed |  |
| **2** | The learner **clearly communicated that the patient needs follow up labs within the week** | | □  Not observed | □  Observed |  |
| **3** | The learner clearly **communicated the patient needs an in-person follow up appointment in a week** | | □  Not observed | □  Observed |  |
| **4** | The learner acknowledged **social determinants of health** and inquired about any barriers to care (e.g., asking about transportation access or expenses for labs) | | □  Not observed | □  Observed |  |

| **COMPETENCY SCORING** | |
| --- | --- |
| **Write in the number of the number of tasks observed complete** | **Score** |
| **Scoring: Not yet entrustable 0\| Approaching entrustment 1 – 2 \| Entrustable 3 – 4** |  |
