# Supplementary material for: How to Conduct Usability Testing of Medical Education Simulations: Methods Tutorial Using a Telemedicine Simulation for Maternal Care
Source: JMIR Med Educ. 2026 Sep 9;12:e102697. doi: 10.2196/102697 (PMC13601883; doi:10.2196/102697)
Supplement: Supplementary file 11 [file mededu_v12i1e102697_app10.docx]

## Appendix 10: Printed Telemedicine Memory Aid
